# Mammalian mitochondrial RNAs are degraded in the mitochondrial intermembrane space by RNASET2

**DOI:** 10.1007/s13238-017-0448-9

**Published:** 2017-07-20

**Authors:** Peipei Liu, Jinliang Huang, Qian Zheng, Leiming Xie, Xinping Lu, Jie Jin, Geng Wang

**Affiliations:** MOE Key laboratory of Bioinformatics, Cell Biology and Development Center, School of Life Sciences, Tsinghua University, Beijing, 100084 China

**Keywords:** mitochondria, intermembrane space, ribonuclease, mtRNA, RNA degradation, decay, RNASET2, RNase T2, inner membrane, transport, RNA trafficking

## Abstract

Mammalian mitochondrial genome encodes a small set of tRNAs, rRNAs, and mRNAs. The RNA synthesis process has been well characterized. How the RNAs are degraded, however, is poorly understood. It was long assumed that the degradation happens in the matrix where transcription and translation machineries reside. Here we show that contrary to the assumption, mammalian mitochondrial RNA degradation occurs in the mitochondrial intermembrane space (IMS) and the IMS-localized RNASET2 is the enzyme that degrades the RNAs. This provides a new paradigm for understanding mitochondrial RNA metabolism and transport.

**Electronic supplementary material:**

The online version of this article (doi:10.1007/s13238-017-0448-9) contains supplementary material, which is available to authorized users.

## INTRODUCTION

Mammalian mitochondria contain a small circular genome, which encodes 2 ribosomal RNAs, 22 tRNAs, and 13 essential protein subunits in the OXPHOS pathway (Anderson et al., 1981). The transcription and translation machineries in the mitochondrial matrix require both mitochondrion-encoded RNAs and nucleus encoded protein and RNA factors (Bonawitz et al., 2006; Rubio et al., 2008; Wang et al., 2010). Mitochondrial RNAs (mtRNAs) are first transcribed as polycistronic transcripts from both H-strand and L-strand, and the transcripts are dissected by RNA processing enzymes into individual rRNAs, tRNAs, and mRNAs (Hallberg and Larsson, 2014; Mercer et al., 2011). These rRNAs and tRNAs together with translational factors imported from cytosol then synthesize the OXPHOS proteins from the mRNAs (Hallberg and Larsson, 2014).

Much is understood about mtRNA synthesis and processing (Sanchez et al., 2011; Schafer et al., 2005). By contrast, less attention has been given to understanding how mtRNAs are degraded. Yet, mitochondrial RNA homeostasis is one of the key elements in regulating mitochondrial functions. Mitochondria have to quickly respond to external stimuli and are constantly going through fusion and fission, which is directly linked to their biosynthesis and requires fast changes of their gene expression (Mishra and Chan, 2016). RNA decay adds a new dimension to the regulation network by changing the abundance of transcripts and removing aberrant RNA molecules and intermediates of transcription and processing. The process must be tightly regulated and often is performed by multi-protein complexes (Szczesny et al., 2012).

In yeast, mtRNAs are degraded by a degradosome complex consisting of a helicase Suv3 and an RNase II like protein Dss1 (Dziembowski et al., 1998; Margossian et al., 1996; Szczesny et al., 2013). Suv3 unwinds and feeds RNA substrates to Dss1, which acts as a 3′ to 5′ exoribonuclease that yields nucleoside monophosphates and a four-nucleotide residual core (Malecki et al., 2010).

In comparison, the degradation machinery of mammalian mtRNAs is much less clear. Even though the role of hSuv3p helicase has been demonstrated, the ribonuclease partner has not been identified (Khidr et al., 2008). Human polynucleotide phosphorylase PNPASE has been shown to be involved in mtRNA degradation and appears to colocalize with Suv3 in distinct mitochondrial foci, but the protein is mainly localized in the mitochondrial intermembrane space (IMS) and functions in mitochondrial RNA import from cytosol (Borowski et al., 2013; Chen et al., 2006; Chujo et al., 2012; Sato et al., 2017; Vedrenne et al., 2012; von Ameln et al., 2012; Wang et al., 2010). Whether its function in mitochondrial RNA degradation is that of a ribonuclease has never been proven. Other ribonucleases such as LACTB2 and Endo G have also been identified in mitochondria (Cote and Ruiz-Carrillo, 1993; Levy et al., 2016; Ohsato et al., 2002; Zhou et al., 2016). However, the exact mechanisms of their involvement in mtRNA degradation have never been resolved. It has long been assumed that mitochondrial RNA decay would occur in the mitochondrial matrix as both yeast and human Suv3 proteins were identified in the matrix. Here we show that contrary to the assumption, mtRNA degradation happens in the mitochondrial intermembrane space (IMS) and IMS-localized RNASET2 is the ribonuclease that carries out the degradation.

## RESULTS

### Characterization of in organello mtRNA degradation

**Figure 1:**
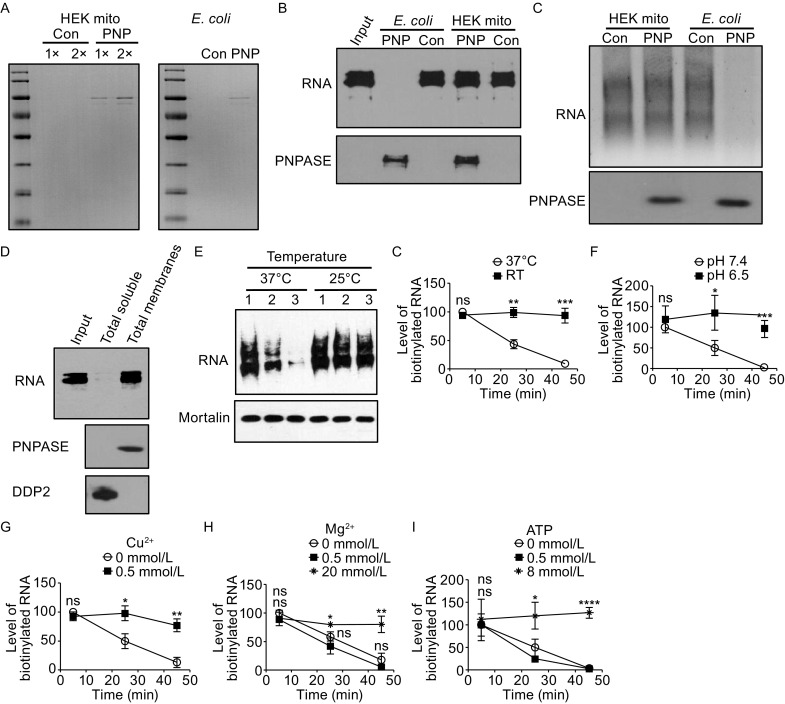
**Characterization of mtRNA degradation using an in organello system**. (A) Coomassie staining of human PNPASE samples purified from HEK293 mitochondria (HEK mito) or *E*. *coli*. For PNPASE purified from HEK mitochondria, two concentrations of samples were loaded. Con: same volume of eluate from cells harboring the empty vector; PNP: eluate from PNPASE-HisPC expressing cells. (B) PNPASE purified from *E*. *coli* or HEK mitochondria was incubated with Biotin-labeled *UCP2* RNA. The lower panel shows immunoblotting of PNPASE. (C) PNPASE purified from *E*. *coli* or HEK mitochondria was incubated with total cytosolic RNA and the samples were resolved on an agarose gel. The lower panel shows immunoblotting of PNPASE. (D) Mitochondria were separated into total soluble and total membrane and the two fractions were examined for ribonuclease activity using biotinylated *UCP2* mRNA as a substrate. Lower panels show Immunoblotting of PNPASE (Membrane) and DDP2 (Soluble). (E) Effect of temperature on in organello mtRNA degradation. Degradation was performed at 37°C (the temperature used for the other experiments if not specified) or 25°C. The three numbers (1, 2, and 3) represent three time points (5 min, 25 min, and 45 min). Top panel on the left shows the remaining labeled mtRNAs. Bottom panel is an immunoblot of mitochondrial protein Mortalin showing the amount of mitochondria taken out at each time point. Right panel shows the quantification of labeled mtRNAs (*n* = 3). (F) Effect of pH on in organello mtRNA degradation. Degradation was performed at pH 7.4 (the pH used for the other experiments if not specified) or pH 6.5. (G) Effect of Cu^2+^ on in organello mtRNA degradation. Two concentrations (0 mmol/L and 0.5 mmol/L) of Cu^2+^ were used. (H) Effect of Mg^2+^ on in organello mtRNA degradation. Three concentrations (0 mmol/L, 0.5 mmol/L, and 20 mmol/L) of Mg^2+^ were used. (I) Effect of ATP on in organello mtRNA degradation. Three concentrations (0 mmol/L, 0.5 mmol/L, and 8 mmol/L) of ATP were used. Statistical comparisons are performed using unpaired *t*-tests (*n* = 3 if not specified); *P < 0.05, **P < 0.01, ***P < 0.001, ****P < 0.0001. Data are presented as mean ± standard error of the mean (s.e.m.)

The identity of the ribonuclease responsible for mammalian mitochondrial RNA degradation had been elusive. It has been shown that human polynucleotide phosphorylase PNPASE and the helicase Suv3 are involved in mtRNA degradation (Borowski et al., 2013; Chujo et al., 2012). However, mammalian PNPASE is localized mainly in the IMS and functions in import of cytosolic RNA into mitochondria (Chen et al., 2006; Sato et al., 2017; Vedrenne et al., 2012; von Ameln et al., 2012; Wang et al., 2010), so it is hard to deduce whether its role in mtRNA degradation is that of a ribonuclease. The ribonuclease activity of mammalian PNPASE has been examined before with the protein expressed in bacteria (Portnoy et al., 2008; Slomovic et al., 2008). We have purified the human PNPASE from both human mitochondria and bacteria as previously described (Wang et al., 2010) and compared the ribonuclease activities. Surprisingly, PNPASE showed no ribonuclease activity in human mitochondria but strong activity when expressed in bacteria (Fig. 1A–C), consistent with a previous report showing mammalian PNPASE has little to no activity in a mammalian system (Sarkar et al., 2005). Total mitochondrial membrane where PNPASE localizes also showed no ribonuclease activity (Fig. 1D). In comparison, strong ribonuclease activity was observed in soluble fraction of the mitochondria that contains no PNPASE (Fig. 1D). Taken together, these results suggest that the function of PNPASE or PNPASE-containing complex in mammalian mtRNA degradation is not that of a ribonuclease. In this report, we will focus on identification of the ribonuclease instead of addressing how mammalian PNPASE ribonuclease activity is inactivated in mitochondria and how PNPASE is involved in mtRNA degradation.

To identify the ribonuclease for mtRNA degradation, an *in vitro* assay is not sufficient, as multiple ribonucleases have been identified in mitochondria (Bruni et al., 2013; Cote and Ruiz-Carrillo, 1993; Levy et al., 2016; Zhou et al., 2016). The ribonuclease that functions directly in mtRNA degradation should have characteristics similar to those of mtRNA degradation. In order to find the ribonuclease, we designed a system that allows in organello characterization of mtRNA degradation and examined the dependency of mtRNA degradation on temperature, pH, ATP, and metal ions. Newly synthesized mtRNAs in isolated mitochondria were labeled with P32 or Biotin. Controls have been performed to show that the isolated mitochondria had no nuclear DNA contamination and the labeled RNAs were mtRNAs in the intact mitochondria as no labeling was observed in the *rho0* mitochondria (Fig. S1A–E). Degradation of the newly synthesized mtRNAs was examined under various chasing conditions. Slower degradation was observed at 25°C than at 37°C (Fig. 1E), and lower pH had an inhibitory effect on the decay (pH 6.5 vs. pH 7.4) (Fig. 1F). The effects of ATP and di-valence metal ions were more complicated: Cu^2+^ had a strong inhibitory effect even at a low concentration (0.5 mmol/L) (Fig. 1G), while Mg^2+^ had little effect on the degradation at a low concentration (0.5 mmol/L) but an inhibitory effect at a higher concentration (20 mmol/L) (Fig. 1H). ATP had a similar effect as that of Mg^2+^, little effect at a low concentration (0.5 mmol/L) but inhibitory at a higher concentration (8 mmol/L) (Fig. 1I).

### mtRNAs are degraded in the mitochondrial intermembrane space (IMS)

**Figure 2:**
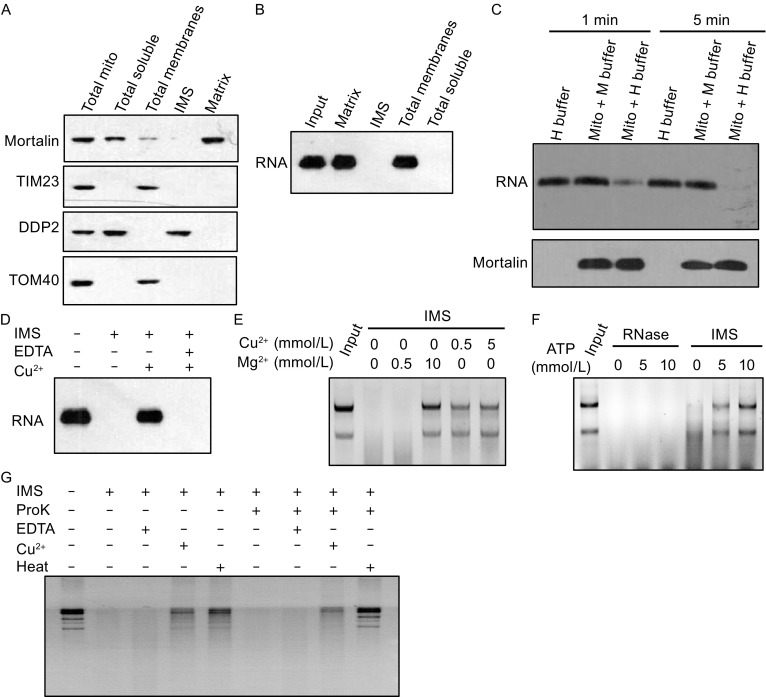
**Characterization of a ribonuclease activity in the mitochondrial IMS**. (A) Immunoblots of total mitochondria and fractions. Mitochondria were separated into four fractions: total soluble (including IMS and Matrix), total membrane, IMS and matrix. Immunoblotting was performed using antibodies for Mortalin (Matrix), TIM23 (Inner membrane), DDP2 (IMS), and TOM40 (Outer membrane). (B) Four mitochondrial fractions were examined for ribonuclease activity using biotinylated *UCP2* mRNA as a substrate. (C) No ribonuclease activity localizes at the outer surface of mitochondrial outer membrane. Isolated mitochondria were resuspended in mitoprep buffer (M buffer) or hypotonic buffer (H buffer) that ruptures the mitochondrial outer membrane. Biotinylated *UCP2* RNA was added to the mixture and incubated at 37°C for 1 min or 5 min before the reaction was terminated. (D) IMS ribonuclease activity was tested for its sensitivity to EDTA (2 mmol/L) and Cu^2+^ (0.5 mmol/L) using biotinylated *UCP2* mRNA as a substrate. (E) IMS ribonuclease activity was tested for its sensitivity to different concentrations of Cu^2+^ (0.5 mmol/L and 5 mmol/L), and Mg^2+^ (0.5 mmol/L and 10 mmol/L) using RNAs purified from isolated mitochondria as substrates. (F) IMS ribonuclease activity and RNaseI were tested for sensitivity to different concentrations of ATP (0 mmol/L, 5 mmol/L, and 10 mmol/L). (G) IMS ribonuclease activity was tested for sensitivity to Proteinase K (ProK), EDTA (2 mmol/L), Cu^2+^ (0.5 mmol/L), and heat (90°C 10 min)

Equipped with the information about mtRNA degradation, we next investigated the possible sub-mitochondrial localization of the mtRNA degradation activity. Using biochemical approaches, we separated mitochondria into four fractions: total soluble, total membranes, IMS, and matrix (Fig. 2A). Biotinylated RNA was used as a substrate to examine the ribonuclease activity of each fraction. Majority of the activity appeared to be in the IMS and the total soluble that includes both IMS and the matrix (Fig. 2B). Since the matrix had little activity, IMS should be where majority of the activity resides (Fig. 2B). To rule out the possibility of a cytosolic contamination, ribonuclease activity was examined using the intact mitochondria. No activity was observed when the mitochondria were incubated with an RNA substrate in the isotonic buffer, but the RNA substrate was quickly degraded when the outer membrane was ruptured using hypotonic buffer, suggesting the outer surface of the purified mitochondria harbors no ribonuclease activity and the IMS activity has no cytosolic contamination (Fig. 2C).

The IMS activity was further characterized and was shown to be inhibited by Cu^2+^ as the in organello mtRNA degradation was, but not by EDTA (Fig. 2D). The activity also degraded total mtRNAs with similar characteristics, and like the in organello mtRNA degradation, its inhibition by Mg^2+^ or ATP was also concentration dependent (Fig. 2E and 2F). In addition, it was inactivated by heating at 90°C, but not by proteinase K treatment (Fig. 2G).

**Figure 3:**
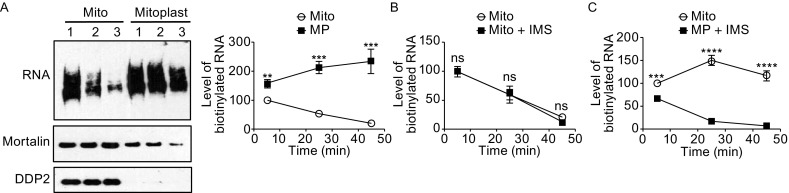
**mtRNAs are degraded in the mitochondrial IMS**. (A) Effect of mitoplasting on in organello mtRNA degradation. The three numbers (1, 2, and 3) represent three time points (5 min, 25 min, and 45 min). Top panel on the left shows the labeled mtRNAs. Middle panel is an immunoblot of mitochondrial matrix protein Mortalin showing the amount of mitochondria taken out at each time point with intact inner membrane. Bottom panel is an immunoblot of mitochondrial IMS protein DDP2 showing the efficiency of mitoplasting. Right panel shows the quantification of the remaining labeled mtRNAs normalized to Mortalin signal (*n* = 3). (B) Effect of adding isolated IMS (0.15 mg/mL) on in organello mtRNA degradation in intact mitochondria. (C) Effect of adding isolated IMS (0.15 mg/mL) on in organello mtRNA degradation in mitoplasts. Statistical comparisons are performed using unpaired *t*-tests (*n* = 3 if not specified); *P < 0.05, **P < 0.01, ***P < 0.001, ****P < 0.0001. Data are presented as mean ± standard error of the mean (s.e.m.)

The localization of a ribonuclease activity in the IMS with characteristics similar to those of in organello mtRNA degradation suggests that mtRNA degradation happens in the IMS. The possibility was investigated using the in organello mtRNA degradation assay. Mitoplasting was performed after the in organello RNA synthesis to rupture the mitochondrial outer membrane, wash away the IMS and rid of some inner membrane associated proteins (Fig. 3A). The inner membrane of the mitoplast did appear more fragile as shown by immunoblotting of the matrix protein Mortalin (Fig. 3A). However, the remaining mitoplast with matrix still protected by the inner membrane appeared to have its mtRNA degradation stopped (Fig. 3A). The newly synthesized mtRNA level actually increased to about 1.5 fold of its original level, suggesting that even under the chase condition there was active mtRNA synthesis and the degradation observed with the intact mitochondria was a result of the balance being tipped towards degradation (Fig. 3A). More interestingly, the stopped mtRNA degradation in the mitoplast was rescued when IMS was added into the degradation buffer and the addition of IMS had no effect on the intact mitochondria (Fig. 3B and 3C). Taken together, these results provide compelling evidence that mtRNA degradation happens in the mitochondrial IMS.

### Identification of an IMS-localized ribonuclease RNASET2

**Figure 4:**
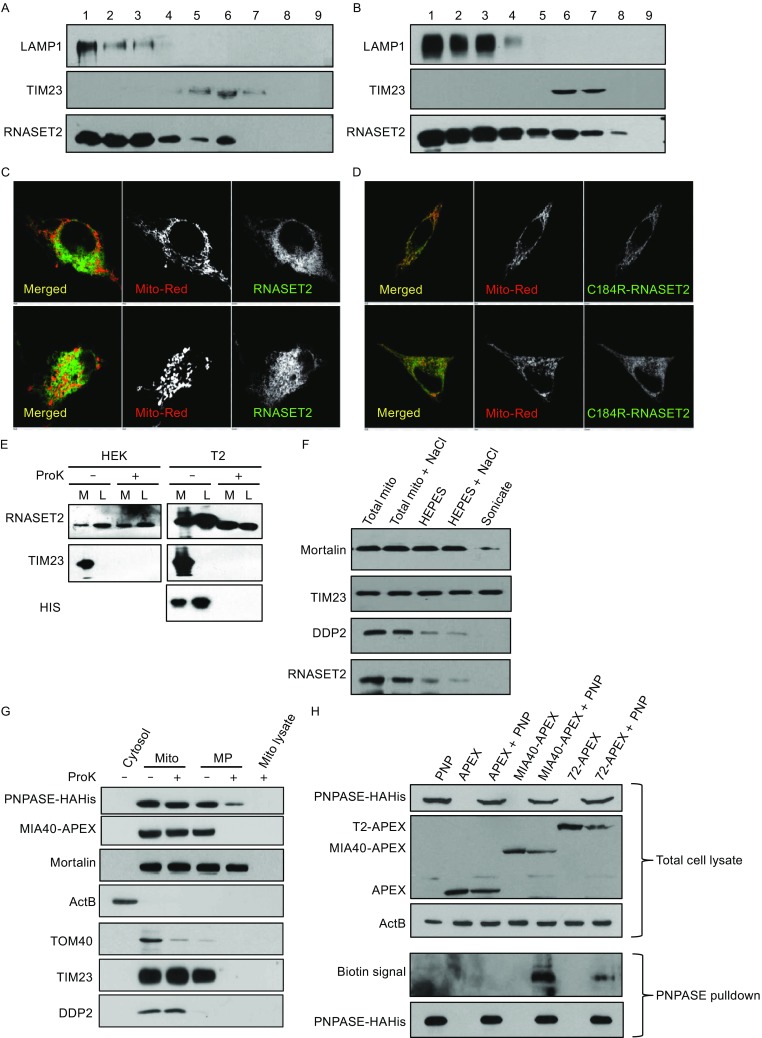

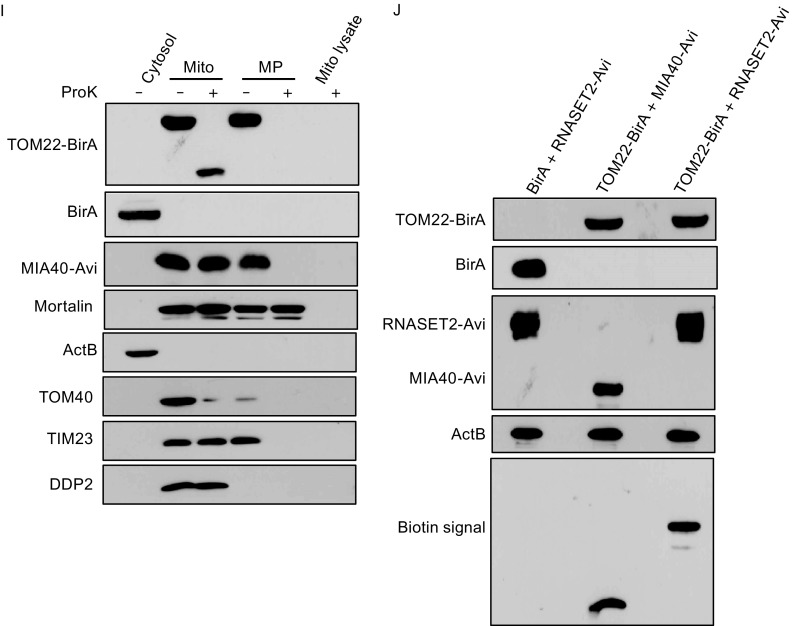
**RNASET2 localizes in mitochondrial IMS**. (A) Immunoblots of different fractions from Optiprep gradient centrifugation of crude HeLa mitochondria with antibodies for lysosomal marker LAMP1, mitochondrial marker TIM23 and RNASET2. (B) Immunoblots of different fractions from Optiprep gradient centrifugation of crude HEK mitochondria overexpressing tagged RNASET2. (C) Confocal microscopy of HeLa cells expressing GFP tagged RNASET2. MitoTracker red (Mito-Red) was used for mitochondrion staining. (D) Confocal microscopy of HeLa cells expressing GFP tagged RNASET2 mutant (C184R). (E) Immunoblots of mitochondrial (M) lysates and lysosomal (L) lysates from control HEK cells or RNASET2-overexpressing cells (T2) with or without proteinase K (ProK) treatment. TIM23 was used as a positive control for Proteinase K treatment. HIS antibody was used to detect the integrity of the tag on overexpressed RNASET2. (F) Immunoblots of mitochondria after undergoing different treatments that exposed different sub-mitochondrial compartments: NaCl (0.3 mol/L) wash to rid the membrane of electrostatically associated proteins, hypotonic treatment to rupture the outer membrane (HEPES), or sonication to rupture both the outer and the inner membrane. Antibodies for Mortalin (matrix), TIM23 (membrane), and DDP2 (IMS) were used. (G) Immunoblots of the cytosol, mitochondria (Mito), mitoplasts (MP, mitochondria with outer membrane ruptured), or mitochondrial lysate with (+) or without (−) proteinase K treatment. PNPASE-HisPC and MIA40-APEX-HAHis (Mia40-Apex) were detected with anti-His antibody. ActB: cytosol; Mortalin: mitochondrial matrix; TOM40: mitochondrial outer membrane; TIM23: mitochondrial inner membrane; DDP2: mitochondrial intermembrane space. (H) Biotinylation of PNPASE by RNASET2-APEX. Top three panels are immunoblots of total lysates from cells expressing PNPASE-HAHis (PNP), APEX-HisPC (APEX), Mia40-APEX-HisPC (MIA40-APEX), RNASET2-APEX-HisPC (T2-APEX) or PNPASE-HAHis together with one of the APEX constructs. Anti-His antibody was used to compare the relative expression levels of the APEX and the fusion proteins (all containing a His tag). Bottom two panels are biotin detection blot and an immunoblot of the PNPASE-HAHis pulldown samples from mitochondria of the six strains listed. (I) Immunoblots of the cytosol, mitochondria (Mito), mitoplasts (MP), or mitochondrial lysate with (+) or without (−) proteinase K treatment. BirA-HAHis (BirA), TOM22-BirA-HAHis (TOM22-BirA), and MIA40-Avi-HisFlag (Mia40-Avi) were detected with anti-His antibody. (J) Biotinylation of RNASET2-Avi-HisFlag (RNASET2-Avi) by TOM22-BirA-HAHis (TOM22-BirA). Upper four panels are immunoblots of total lysates from cells expressing BirA-HAHis with RNASET2-Avi-HisFlag, TOM22-BirA-HAHis with MIA40-Avi-HisFlag, or TOM22-BirA-HAHis with RNASET2-Avi-HisFlag. Anti-His antibody was used to detect BirA and the fusion proteins (all containing a His tag), and anti-Flag antibody was used to detect Avi fusion proteins. Bottom panel is a biotin detection blot

With the localization of the ribonuclease for mammalian mtRNA degradation confirmed, we next tried to identify the activity (Fig. S2A and S2B). Based on the mass-spectrometry results of the purified samples (Tables S1 and S2), we singled out RNASET2 for further testing. Mammalian RNASET2s are endoribonucleases that are targeted for secretion and also to lysosomes (Acquati et al., 2011; Luhtala and Parker, 2010). Optiprep gradient fractionation showed HeLa cells have a distinct mitochondrial pool of endogenous RNASET2 (Fig. 4A). Overexpression of RNASET2 did not cause a dramatic change of the gradient centrifugation profile (Fig. 4B), indicating a similar localization of the tagged protein to that of the endogenous RNASET2. Colocalization of GFP-tagged RNASET2 and mitoTracker Red was also observed using confocal microscopy (Fig. 4C). A mutation (C184R) that disrupts lysosomal localization led to a better colocalization of RNASET2 with mitochondria (Fig. 4D), suggesting a dynamic balance among different subcellular pools.

Since the IMS ribonuclease activity is insensitive to proteinase K treatment, we tested whether RNASET2 could be digested by proteinase K. Consistent with the IMS result, both the endogenous RNASET2 and the tagged version were resistant to proteinase K digestion, while TIM23 was readily degraded by the protease (Fig. 4E). The tagged version ran lower on SDS-PAGE after proteinase K treatment simply because the tag was digested by the protease as shown by a lack of immunoblotting signal for the tag after the treatment (Fig. 4E). Because RNASET2 was proteinase K insensitive, Mitoplasting and NaCl wash instead of the traditional Mitoplasting and protease treatment was performed to examine the sub-mitochondrial localization of the endogenous RNASET2. Endogenous RNASET2 showed a fractionation profile similar to that of an IMS protein DDP2 but different from those of a membrane protein BAP37 and a matrix protein Mortalin, suggesting an IMS localization of RNASET2 within mitochondria (Fig. 4F).

To rule out the possibility that the localization of RNASET2 in mitochondria we observed was due to contamination from other cellular compartments, we took advantage of a new technique called proximity-based labeling, which has been used to overcome the problem of fractionation contamination (Han et al., 2017; Kim and Roux, 2016; Williams et al., 2014). Ascorbate peroxidase (APEX) biotinylates proteins in close proximity when biotin-phenol and H_2_O_2_ are added to the medium of live cells (Jan et al., 2014). APEX was used to tag RNASET2 and a mitochondrial IMS protein MIA40. Subcellular fractionation and mitoplasting with or without proteinase K treatment was performed to ensure that APEX and other tags had no effect on the localization of the proteins (Fig. 4G). Biotinylation of mitochondrial IMS protein PNPASE was observed only when MIA40-APEX or RNASET2-APEX was expressed but not when cytosolic APEX was expressed at a similar level, further proving a mitochondrial localization of RNASET2 (Fig. 4H). This technique, however, lacks specificity; so another proximity labeling approach was adopted. Biotin ligase (BirA) biotinylates Avi-tagged proteins when they are in close proximity (Roux et al., 2012). BirA was used to tag a mitochondrial outer membrane protein TOM22 with its C-terminus facing the IMS and Avi was used to tag RNASET2 and MIA40. Subcellular fractionation and mitoplasting with or without proteinase K treatment was again performed to ensure that BirA, Avi, and other tags had no effect on the localization of the proteins and that BirA on the C-terminus of TOM22 is in the IMS (Fig. 4I). TOM22-BirA but not cytosolic BirA quickly biotinylated both MIA40-Avi and RNASET2-Avi, when Biotin was added to the medium, proving once again RNASET2 is localized in the mitochondrial IMS (Fig. 4J).

### RNASET2 functions in mtRNA degradation and indirectly affects mtRNA synthesis

**Figure 5:**
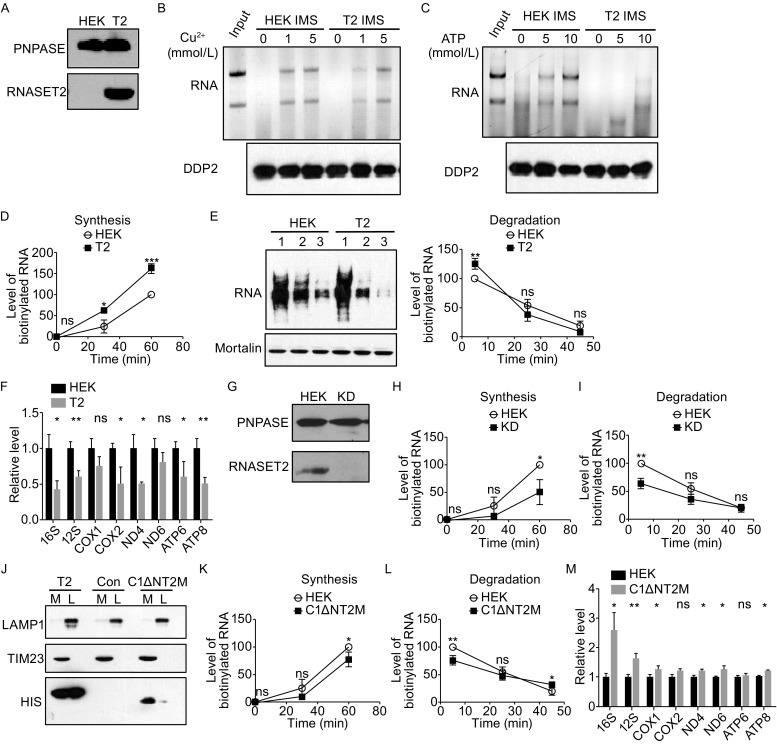
**RNASET2 functions in mtRNA degradation**. (A) Immunoblots of HEK and RNASET2-overexpressing (T2) cell lysates. PNPASE was used as a loading control. (B) IMS was isolated from HEK mitochondria or RNASET2-overexpressing (T2) mitochondria and the ribonuclease activity was tested for its sensitivity to different concentrations of Cu^2+^ (1 mmol/L and 5 mmol/L). Bottom panel is an immunoblot of IMS protein DDP2 showing the relative amount of IMS used for each *in vitro* degradation assay. (C) IMS was isolated from HEK mitochondria or RNASET2-overexpressing (T2) mitochondria and the ribonuclease activity was tested for its sensitivity to different concentrations of ATP (5 mmol/L and 10 mmol/L). (D) In organello mtRNA synthesis in control mitochondria (HEK) and mitochondria overexpressing RNASET2 (T2). (E) In organello mtRNA degradation in control mitochondria (HEK) and mitochondria overexpressing RNASET2 (T2). (F) qRT-PCR of mRNAs in HEK and RNASET2-overexpressing cells (T2) after H_2_O_2_ treatment for 2 h. (G) Immunoblots of HEK or RNASET2-knockdown (KD) cell lysates. PNPASE was used as a loading control. (H) In organello mtRNA synthesis in control mitochondria (HEK) and RNASET2-knockdown mitochondria (KD). (I) In organello mtRNA degradation in control mitochondria (HEK) and RNASET2-knockdown mitochondria (KD). (J) Immunoblots of mitochondria (M) and lysosomes (L) isolated from RNASET2-overexpressing cells (T2), control HEK cells (Con), and cells expressing C1-ΔN-RNASET2 (C65R, C118R) mutant (C1ΔNT2M). LAMP1 was used as a lysosomal marker, and TIM23 as a mitochondrial marker. (K) In organello mtRNA synthesis in control mitochondria (HEK) and mitochondria expressing C1-ΔN-RNASET2 (C65R, C118R) mutant (C1ΔNT2M). (L) In organello mtRNA degradation in control mitochondria (HEK) and mitochondria expressing C1-ΔN-RNASET2 (C65R, C118R) mutant (C1ΔNT2M). (M) qRT-PCR of mRNAs in HEK and C1-ΔN-RNASET2 (C65R, C118R) mutant expressing cells (C1ΔNT2M) after H_2_O_2_ treatment for 2 h. Statistical comparisons are performed using unpaired *t*-tests (*n* = 3 if not specified); *P < 0.05, **P < 0.01, ***P < 0.001, ****P < 0.0001. Data are presented as mean ± standard error of the mean (s.e.m.)

To verify the involvement of RNASET2 in mtRNA degradation, it was overexpressed in the HEK293 cells (Fig. 5A). IMS was isolated from the RNASET2-overexpressing mitochondria and the control mitochondria, and the ribonuclease activity examined. Compared to the control IMS, RNASET2-overexpressing IMS was less sensitive to Cu^2+^ or ATP inhibition (Fig. 5B and 5C). The in organello mtRNA synthesis was faster for the RNASET2-overexpressing mitochondria (Fig. 5D) and the in organello mtRNA degradation rate was also higher (Fig. 5E). Under *in vivo* condition that was partially inhibitory to mitochondrial RNA synthesis (H_2_O_2_ treatment), RNASET2 overexpression led to a significant decrease of the mtRNA levels (Fig. 5F). RNASET2 knockdown with shRNAs showed opposite effects both on in organello mtRNA synthesis and degradation with mtRNA synthesis and degradation rates decreased in the knockdown mitochondria (Fig. 5G and 5H). The effects were not restricted to human cells, as overexpression of mouse RNASET2 in a mouse cell line TM6 also led to an increase of both in organello mtRNA synthesis and degradation rates (Fig. S3A–C).

Since RNASET2 is a protein with multiple subcellular localizations, the effects of overexpression and knockdown on mtRNA degradation could be indirect and be due to changes of its functions in other cellular compartments. To rule out the possibility, we fused an enzymatically dead mutant (C65R, C118R) without the N-terminal 24 amino acids to the targeting sequence of cytochrome C1 that targeted the protein to the IMS (C1ΔNT2M). Unlike the wild-type RNASET2, C1ΔNT2M localizes predominately in the mitochondria (Fig. 5J). This enzymatically dead mutant, when targeted to the IMS, acting like a dominant negative mutant, had similar effects on the in organello mtRNA synthesis and degradation as those of RNASET2 knockdown (Fig. 5K and 5L). Under *in vivo* condition that was inhibitory to mitochondrial RNA synthesis, C1ΔNT2M expressing mitochondria contained significantly higher levels of mitochondrial rRNAs and showed less structural defects (Figs. 5M and S4). The mild effect of C1ΔNT2M on mitochondrial mRNA levels is possibly due to the feedback down-regulation of mtRNA synthesis, as there is no efficient approach to quickly turn off mtRNA synthesis without dramatically affecting other cellular processes (Fig. 5K). Taken together, these data suggest the mitochondrial pool of RNASET2 indeed functions in mtRNA degradation and its ribonuclease activity is required.

### Purified RNASET2 has characteristics similar to those of in organello mtRNA degradation

**Figure 6:**
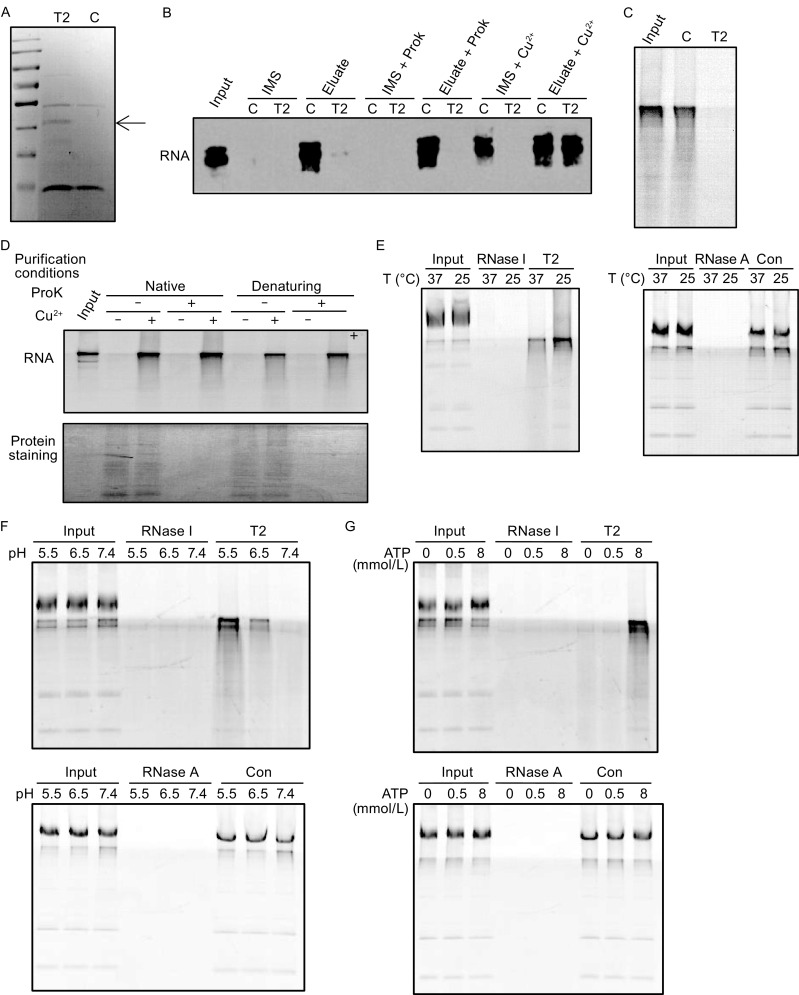

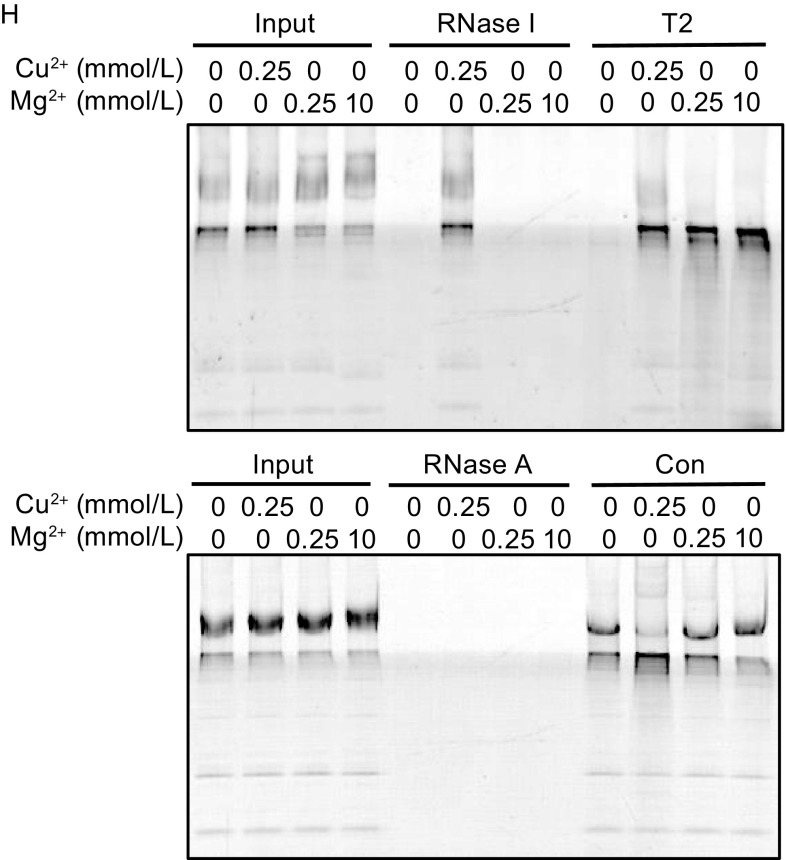
**Characterization of RNASET2 purified from HEK mitochondria**. (A) Dual-tag purification of RNASET2 (His and HA). Purification was performed using IMS from control HEK cells (C) or RNASET2-overexpressing cells (T2) under native condition. (B) Ribonuclease activity were examined in IMS samples and the purification samples (Eluate) from control HEK cells (C) or RNASET2-overexpressing cells (T2) using biotinylated *UCP2* mRNA as a substrate. The sensitivity of these activities to Cu^2+^ (0.5 mmol/L) and proteinase K (ProK) was also tested. (C) RNASET2 was purified from RNASET2-overexpressing mitochondria under denaturing condition and checked for ribonuclease activity using RNA purified from isolated mitochondria as substrates; C (control pulldown from HEK mitochondria) and T2 (RNASET2). (D) RNASET2s purified under native conditions and denaturing conditions had the same responses to proteinase K (ProK) and Cu^2+^ treatment. Lower panel is a coomassie staining gel of mitochondrial lysates as a positive control for proteinase K treatment. (E) Effect of temperature on RNASET2 purified from HEK mitochondria, RNaseI, and RNaseA (50 ng). Degradation was performed at 37°C (the temperature used for the other experiments if not specified) or 25°C. RNAs purified from isolated mitochondria were used as substrates. Con (control pulldown from HEK mitochondria). (F) Effect of pH on RNASET2 purified from HEK mitochondria, RNaseI, and RNaseA. Degradation was performed at pH 7.4 (the pH used for the other experiments if not specified), pH 6.5 or pH 5.5. (G) Effect of ATP on RNASET2 purified from HEK mitochondria, RNaseI and RNaseA. (H) Effect of Mg^2+^ and Cu^2+^ on RNASET2 purified from HEK mitochondria, RNaseI and RNaseA

A dual tagged human RNASET2 was expressed in HEK293 cells and purified under native conditions from the IMS (Fig. 6A). The purified proteins contained a ribonuclease activity that was also insensitive to proteinase K treatment and was inhibited by Cu^2+^ (Fig. 6B). RNASET2 purified under denaturing conditions showed same characteristics as that purified under native conditions, suggesting the activity came from RNASET2 itself instead of interacting proteins (Fig. 6C and 6D). Conditions tested on in organello mtRNA degradation assay were thoroughly tested on the purified protein. The responses of the protein to temperature, pH, ATP, Cu^2+^ and Mg^2+^, and those of the in organello system showed a striking match, while other ribonucleases showed vastly different responses: RNaseI only responded to Cu^2+^ treatment with its activity inhibited, and RNaseA was insensitive to all the treatments (Fig. 6E–H).

The response of purified RNASET2 to pH is of great interest. Protozoan RNASET2s are mostly acidic ribonucleases (Irie, 1999), but the human RNASET2 appears to favor more of a neutral pH (Fig. 6F). This change could be due to changes of the amino acid sequence or post-translational modification in mammalian cells. To find the reason behind the change, human RNASET2 was expressed in bacteria and the protein was purified under denaturing conditions (Fig. S5A). Again, the purified protein was insensitive to proteinase K treatment (Fig. S5B). The responses to temperature, pH, ATP, Cu^2+^ and Mg^2+^ were also identical to the protein purified from mammalian cells (Fig. S5C–F), suggesting the activity of human RNASET2 is intrinsically different from its protozoan counterparts.

Another IMS localized nuclease is Endo G (Cote and Ruiz-Carrillo, 1993; Ohsato et al., 2002; Zhou et al., 2016). To examine whether it has similar characteristics as in organello mitochondrial RNA degradation and can potentially be also involved in the process, we purified human Endo G from HEK mitochondria and *E*. *coli* (Fig. S6A–E). Unlike RNASET2, Endo G was sensitive to proteinase K and the responses to the conditions tested were vastly different from those of RNASET2 or in organello mitochondrial RNA degradation. Thus, a direct involvement of Endo G on mitochondrial RNA degradation seems unlikely. That the human RNASET2 purified from mammalian cells and bacteria showed responses to so many conditions almost identical to those of the in organello mtRNA degradation together with its IMS localization further verified it as the enzyme responsible for mtRNA degradation.

## DISCUSSION

For decades, the identity of the ribonuclease responsible for degradation of mammalian mitochondrial RNAs (mtRNAs) had been a great mystery. Our attempt at unraveling this mystery uncovered a mitochondrial IMS-localized ribonuclease activity that degrades mtRNAs and is sensitive to many cellular conditions such as pH and Ca^2+^. We have done extensive studies to rule out the possibility that the action on the substrates was due to a cytosolic contamination or a mitochondrial leak. Localization of such a ribonuclease activity in the mitochondrial IMS guarantees a faster change of mitochondrial biogenesis and quick regulation of mitochondrial functions in response to signals outside of mitochondria.

At first glance, degradation of mtRNA in the IMS instead of the matrix where transcription and translation occur seems a little radical. However, this is not the first time a nuclease is reported in the IMS. REXO2, a 3’ to 5’ exonuclease specific for small oligomers also has an IMS localization (Bruni et al., 2013). It could act on oligonucleotides yielded by RNASET2 and recycle the NTPs for new mtRNA synthesis. Another example is Endo G, a nuclease reported to primarily localize in mitochondrial IMS and function on degradation of both nuclear and mitochondrial DNA under special circumstances (Cote and Ruiz-Carrillo, 1993; Ohsato et al., 2002; Zhou et al., 2016). Proteinase K insensitivity of RNASET2 is also a surprising finding. However, it is not a unique feature. Same characteristic is shared by ribonucleases in *Leishmania tarentolae* mitochondria (Alfonzo et al., 1998; Simpson et al., 1992).

Degradation of mtRNAs in the mitochondrial IMS means there are active transports of RNAs from the matrix across the mitochondrial inner membrane. Both mitochondrial RNA import and export have been previously reported. Mitochondria import a wide array of RNAs from the cytosol, including tRNAs, 5S rRNA and other non-coding RNAs, and the import pathways have been partially characterized (Chang and Clayton, 1989; Duchene et al., 2009; Mercer et al., 2011; Noh et al., 2016; Smirnov et al., 2011; Wang et al., 2010; Zhang et al., 2014). Mitochondrion-derived MitomiRs could also be exported to cytosol to function in post-transcriptional regulation of gene expression (Bienertova-Vasku et al., 2013; Duarte et al., 2015). These existing import and export pathways could potentially be the pathways for substrate delivery to the IMS ribonuclease activity. More studies are needed to understand substrate selection and the transport pathways.

The mammalian IMS ribonuclease activity shown here is quite different from yeast mtRNA degradosome and bacterium degradosome (Dziembowski et al., 1998; Miczak et al., 1996; Szczesny et al., 2012). Yeast degradosome has its ribonuclease component Dss1 in the matrix, and bacterium degradosome ribonuclease PNPASE is absent in yeast (Miczak et al., 1996; Wang et al., 2010). Mammalian PNPASE resides in mitochondrial IMS. It has been reported to be involved in mtRNA degradation and processing (Clemente et al., 2015; Daoud et al., 2012), but based on our results, a direct role as mtRNA ribonuclease seems unlikely. We have shown that mammalian mitochondrial membranes have no apparent ribonuclease activity (Fig. 2A and 2B), and PNPASE, a mitochondrial membrane bound protein, has no ribonuclease activity (Fig. 1A–C). A known role for mammalian PNPASE is importing nucleus-encoded RNAs into mitochondria (Sato et al., 2017; Vedrenne et al., 2012; von Ameln et al., 2012; Wang et al., 2010), so it could have trafficking role in substrate transport for the IMS ribonuclease activity. Evidence that mammalian PNPASE is not the ribonuclease for mtRNA degradation but could be involved in their transport also comes from RNAi silencing of PNPASE expression. In some cases, a decrease of mtRNA level was observed; while in others, there were no significant changes (Slomovic and Schuster, 2008).

An important question that arose from this study is how the RNA degradation activity is coordinated with the transcriptional activity in mitochondria. Mitochondria appear to be capable of quickly up-regulate transcriptional activity when the IMS RNA degradation activity is up, and vice versa, hence maintaining relatively stable RNA levels (Fig. 5). A signaling pathway or pathways are clearly needed for such regulation. The responses of both RNASET2 activity and in organello mitochondrial RNA degradation to conditions such as ATP and pH also suggest coupling and a feedback circuit between mitochondrial RNA degradation and ETC activity/ATP synthesis.

Interestingly, RNASET2 has been shown to be involved in cancer suppression and loss of function mutation causes cystic leukoencephalopathy (Acquati et al., 2011; Henneke et al., 2009). An enzymatic dead mutant without the N-terminus also has tumor suppression activity (Nesiel-Nuttman et al., 2015). However, we have shown that truncation of the N-terminus led to mislocalization of the protein, so possible involvement of its ribonuclease activity in tumor suppression has not been rule out yet. Since mammalian RNASET2 has little ribonuclease activity at low pH, the function of lysosomal pool remains to be elucidated. More work is also needed to understand its physiological roles.

## MATERIALS AND METHODS

### Cell lines

Cell lines used include HEK293, HeLa, TM6, and stable cell lines generated on these cell lines. See Supplemental Experimental Procedures for details.

### Plasmids

Plasmids used include *PQCXIP-RNASET2-HAHisPC*, *PQCXIP-C1-ΔN-RNASET2* (*C65R*, *C118R*)*-HAHisPC*, *PQCXIP-RNASET2-GFP*, *PQCXIP-RNASET2* (*C184R*)*-GFP*, *PET28A-RNASET2-HisPC, PQCXIP-EndoG-HAHis*, *PET28A-EndoG-HAHis*, *PQCXIP-APEX-HisPC*, *PQCXIP-RNASET2-APEX-HisPC*, *PQCXIP-MIA40-APEX-HisPC*, *PQCXIP-PNPASE-HAHis, PQCXIP-MIA40-Avi-HisFlag, PQCXIP-RNASET2-Avi-HisFlag*, and *PQCXIP-TOM22-BirA-HAHis*. See Supplemental Experimental Procedures for construction details.

### Isolation of crude mammalian mitochondria and cytosol

Cells were washed once with PBS buffer, resuspended in ice-cold mitoprep buffer (0.225 mol/L manifold, 0.075 mol/L sucrose, and 20 mmol/L HEPES pH 7.4), and broken in a glass-Teflon homogenizer on ice with 30 strokes. Nuclei and unbroken cells were pelleted at 700 ×*g* for 5 min, and the homogenization repeated once. Supernatants from both times were centrifuged again at 700 ×*g*. Crude mitochondria were pelleted from second-round supernatants at 11,000 ×*g* for 5 min, washed once with mitoprep buffer and resuspended in mitoprep buffer of desired volume for further use. Post-mitochondrial supernatant was spun at 21,000 ×*g* for 10 min and the supernatant was collected as cytosol.

### Sub-mitochondrial fractionation

Hypotonic treatment was performed by incubating mitochondria for 20 min on ice by diluting mitoprep buffer with 10 volumes of 20 mmol/L HEPES pH 7.4 with one gentle vortexing at 10 min. Mitoplasts were separated from IMS by centrifugation at 15,000 ×*g* for 4 min. Salt wash was performed by adding 300 mmol/L of NaCl (from 5 mol/L stock) into mitoprep buffer (in cases of intact mitochondria) or mitoplasting mixture (in cases of mitoplasts) after 20 min on ice for another 5 min. Mitochondrial matrix was isolated by sonicating the salt-washed mitoplast in mitoprep buffer and separating the soluble fraction (matrix) from membrane by 10 min 21,000 ×*g* centrifugation. Total soluble was isolated by sonicating mitochondria in mitoprep buffer and separating the soluble fraction (total soluble) from membranes by 10 min 21,000 ×*g* centrifugation. The pellet was washed twice with mitoprep buffer and used as total membranes.

Mitoplasting after in organello mtRNA synthesis was carried out by proteinase K treatment. 500 μg mitochondria in 200 μL in organello synthesis mixture were pelleted by 12,000 ×*g* 4 min spin, washed with 1 mL mitoprep buffer, and resuspended in 300 μL mitoprep buffer with 2 μg proteinase K. The mixture was incubated on ice for 15 min with one vortexing at 8 min. 1 mL mitoprep buffer was added after the incubation and mitoplasts were pelleted at 15,000 ×*g* for 4 min, washed once with 1 mL mitoprep buffer, and proceeded to in organello degradation. Control mitochondria samples skipped the last two spins, as spinning and resuspending at this stage ruptures the outer membrane. This new method was used to ensure degradation of some inner membrane bound proteins that might be involved in mtRNA export from the matrix.

### *In vitro* degradation assay

*In vitro* degradation assay for PNPASE was performed as previously described (Wang et al., 2010). All the other assays were performed in 20 mmol/L HEPES pH 7.4 at 37°C for 10 min if not otherwise specified. Mitochondrial lysates that contain membrane fractions had 0.5% Triton X. For experiments with membrane samples, equal amount of Triton X was added into all samples, normally the final concentration not exceeding 0.1% because of dilution. ~2 μg of IMS, ~2 μg of Matrix or ~10 μg of membrane was used for each 20 μL reaction. For purified protein samples, RNaseI (Thermo) and RNaseA (Thermo), 50 ng was used for each reaction. Reactions with bacterial samples were incubated for 20 min instead of 10 min. Substrates included 1 ng biotinylated *UCP2*, and 300 ng mtRNA. For effects of metal ions on the degradation, different concentrations of MgCl_2_, CuSO_4_, MnCl_2_, or ZnCl_2_ were used. Reaction was stopped by adding equal volume of SDS-Urea-EDTA buffer (2× SDS loading buffer with 8 mol/L urea and 15 mmol/L EDTA) and incubating at 90°C for 5 min. Samples were cooled to room temperature and 0.5 μg of proteinase K was added for a 5 min incubation at 37°C. Biotinylated samples were run on SDS-PAGE, transferred to a nylon membrane, and detected with nucleic acid detection kit (Thermo). mtRNA samples were run either on SDS-PAGE or agarose gels and stained with EtBr.

### In organello RNA synthesis

In organello RNA synthesis was performed in 200 μL mitoprep buffer containing 4 mmol/L ATP pH 7.4, 20 mmol/L succinate, 1 mmol/L CaCl_2_ and 1 μL Biotin RNA Labeling Mix (Roche) with 500 μg mitochondria at 37°C. For each time point (0 min, 30 min, and 60 min), 60 μL reaction mix was taken out and mitochondria were pelleted at 18,000 ×*g* for 2 min. Pellets were stored at −80°C for at least 15 min before next preparation step. For loading, samples were taken out of −80°C, quickly dissolved in 30 μL SDS-Urea-EDTA buffer (SDS loading buffer with 8 mol/L urea and 15 mmol/L EDTA) preheated to 90°C, and incubated at 90°C for 5 min. Samples were then cooled to room temperature and 0.5 μg of proteinase K was added for a 5 min incubation at 37°C. Biotinylated samples were run on SDS-PAGE, transferred to a nylon membrane (400 mA for 1.5 h), and detected with nucleic acid detection kit (Thermo). These synthesis conditions are less inhibitory to mtRNA degradation, and were used for the in organello degradation assays that examined their responses to mitoplasting, temperature, pH, ATP, and metal ions.

For comparison of in organello synthesis between different cell lines, more inhibitory synthesis conditions were used to ensure the yield of newly synthesized RNA more representing the real synthesis rate. Therefore, in organello mtRNA synthesis were carried out in buffer containing 4 mmol/L ATP pH 7.4, 20 mmol/L succinate, 0.5 mmol/L CaCl_2_, 10 mmol/L MgCl_2_, and 1 mg/mL HEK cytosol.

### In organello mtRNA degradation

For in organello degradation assays that examined their responses to mitoplasting, temperature, pH, ATP, and metal ions, 500 μg mitochondria first underwent mtRNA synthesis in 200 μL mitoprep buffer containing 4 mmol/L ATP pH 7.4, 20 mmol/L succinate, 1 mmol/L CaCl_2_, and 1 μL Biotin RNA Labeling Mix (Roche) at 37°C for 45 min. Mitochondria were pelleted at 12,000 ×*g* for 4 min at 4°C, washed with 1 mL ice cold mitoprep buffer, resuspended in 150 μL mitoprep buffer, and incubated on ice for 15 min with one vortexing at 8 min. 50 μL ice cold cocktail containing 4 mmol/L UTP, 40 mmol/L Ca^2+^, 1 μL Ribolock RNase inhibitor (Thermo), and 1 μg RNaseI (Thermo) (original buffer exchanged to mitoprep buffer) in mitoprep buffer was added to the sample (RNase inhibitor was used to eliminate cytosolic interference and RNaseI was used to quickly degrade the RNA from broken mitochondria). The samples were shifted to 37°C. For each time point (0 min, 30 min, and 60 min), 60 μL reaction mix was taken out and mitochondria were pelleted at 18,000 ×*g* for 2 min. Pellets were stored at −80°C for at least 15 min before next preparation step. For loading, samples were taken out of −80°C, quickly dissolved in 30 μL SDS-Urea-EDTA buffer (SDS loading buffer with 8 M urea and 15 mmol/L EDTA) preheated to 90°C, and incubated at 90°C for 5 min. Samples were then cooled to room temperature and 0.5 μg of proteinase K was added for a 5 min incubation at 37°C. Biotinylated samples were run on SDS-PAGE, transferred to a nylon membrane (400 mA for 1.5 h), and detected with nucleic acid detection kit (Thermo).

For comparison of in organello mtRNA degradation between different cell lines, in organello mtRNA synthesis were carried out in buffer containing 4 mmol/L ATP pH 7.4, 20 mmol/L succinate, 0.5 mmol/L CaCl_2_, and 1 mg/mL HEK cytosol for 45 min at 37°C before the following wash and degradation steps. Mitochondria should be isolated from stable cells lines grown to less than 5 doubling times from the initial frozen stocks.

### Biotin-phenol labeling with APEX in live cells

500 μmol/L biotin-phenol (ApexBio) was added to cell medium for 30 min. Then 1 mmol/L of H_2_O_2_ was added and the plates were gently agitated for 1 min. The reaction was quickly quenched by replacing the medium with DPBS containing 5 mmol/L Trolox (abcam) and 10 mmol/L sodium ascorbate (Solarbio). Cells were then washed with the same solution three times. For Ni-NTA (Qigen) and HA bead (Thermo Scientific) enrichment of PNPASE, mitochondria were isolated from the biotin-labeled cells and PNPASE was first purified from the lysate under denaturing condition using Ni-NTA beads and then using HA beads under native conditions according to the manufactures’ instructions.

### *In vivo* biotinylation of Avi-tagged protein by spatially localized biotin ligase (BirA)

HEK293 cells co-transfected with the Avi-tagged protein expressing plasmid and BirA fusion protein expressing plasmid were gown in medium containing 25 μmol/L D-Biotin (Amresco) for 12 h. The cells were harvested, washed with PBS time times, lysed in 1× SDS protein loading buffer, and run on SDS-PAGE for biotin detection.

### Additional procedures

Additional procedures include protein purification, Western blotting, mtRNA isolation, qPCR, Optiprep gradient centrifugation, identification of IMS ribonuclease, fluorescence microscopy and image acquisition, and *in vitro* transcription. See Supplemental Experimental Procedures for details.

## Electronic supplementary material

Below is the link to the electronic supplementary material.

Supplementary material 1 (PDF 1633 kb)
