# Supplementary material for: Mammalian mitochondrial RNAs are degraded in the mitochondrial intermembrane space by RNASET2
Source: Protein Cell. 2017 Jul 20;8(10):735–49. doi: 10.1007/s13238-017-0448-9 (PMC5636749; doi:10.1007/s13238-017-0448-9)
Supplement: Supplementary file 1 — Supplementary material 1 (PDF 1633 kb) [file 13238_2017_448_MOESM1_ESM.pdf]

Mammalian mitochondrial RNAs are degraded in the mitochondrial intermembrane space by RNASET2

Peipei Liu, Jinliang Huang, Qian Zheng, Leiming Xie, Xinping Lu, Jie Jin, Geng Wang*

SUPPLEMENTAL DATA

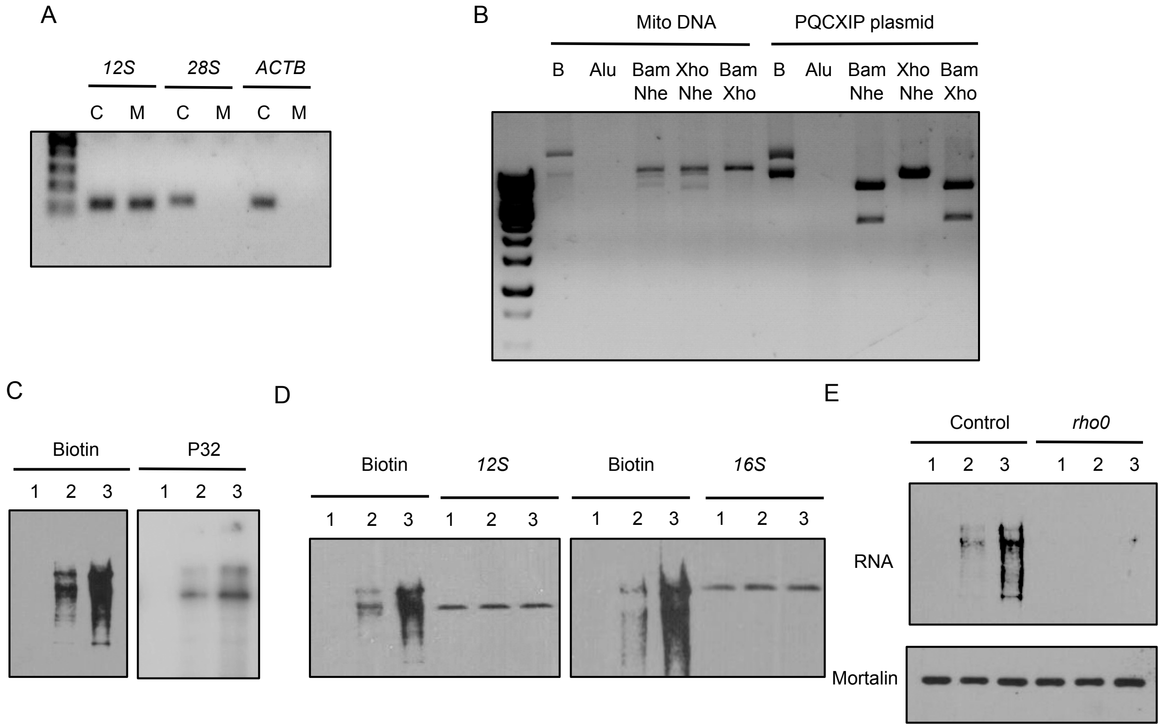

Figure S1. Isolated mitochondria have no nuclear DNA contamination and the two major bands of in organello mitochondrial RNA synthesis are *12S* and *16S* rRNAs.
(A) PCR amplification of DNA isolated from whole cell lysate (C) or mitochondria with primers for mitochondrial *12S* or nuclear *28S* and *ACTB*.
(B) DNA isolated from mitochondria or PQCXIP plasmid was digested with different sets of enzymes, B (buffer), Alu (AluI), Bam (BamHI), Nhe (NheI), Xho (Xho1).
(C) In organello mtRNA synthesis in HEK mitochondria with Biotin labeled NTP or P32 labeled NTP. The three numbers (1, 2 and 3) represent three time points (0 min, 30 min and 60 min)
(D) Side by side comparison of in organello mtRNA synthesis with northern blot of *12S* or *16S* rRNA. Same RNA blot was cut into two halves and one for detection of newly synthesized mtRNA and the other for total *12S* or *16S* rRNA detection using biotin labeled RNA as probes.
(E) In organello mtRNA synthesis in control HEK mitochondria and *rho0* mitochondria. Top panel shows the biotinylated RNAs and the bottom panel is an immunoblot of mitochondrial protein Mortalin.

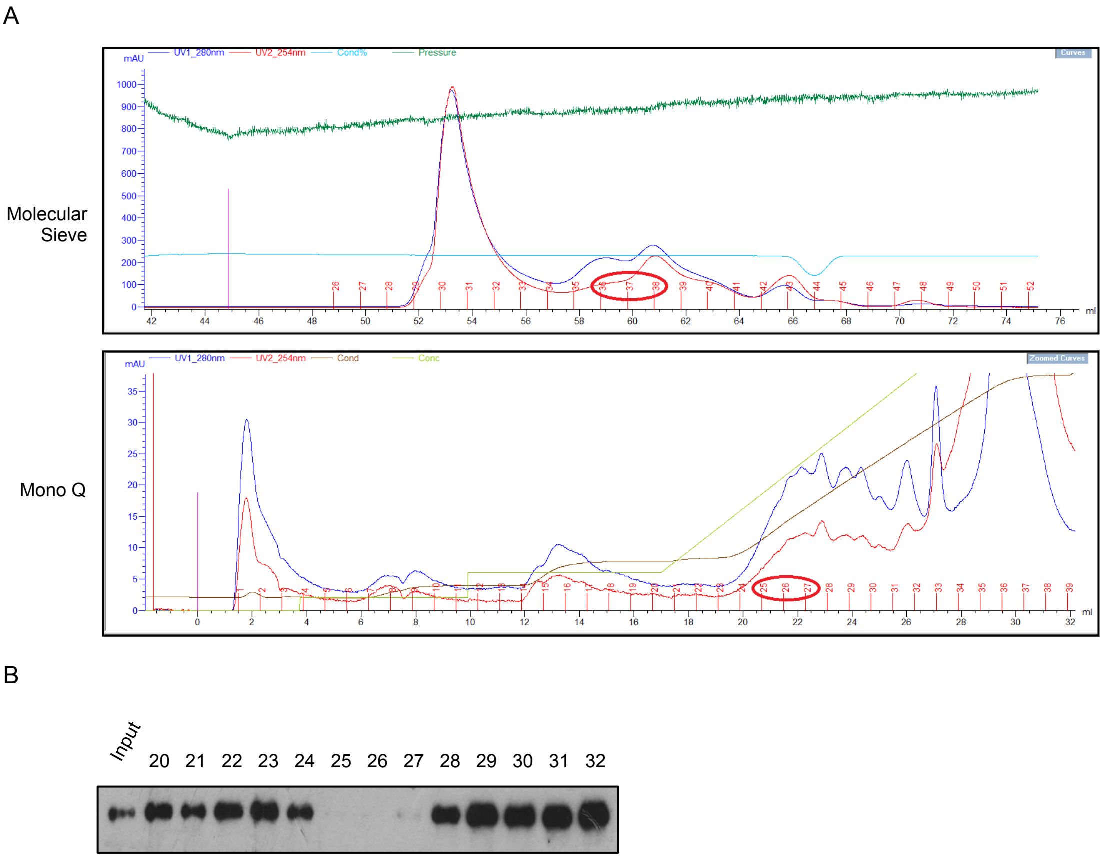

Figure S2. Identification of IMS ribonuclease activity.

(A) Chromatograms from Molecular sieve and Mono Q running of IMS samples. Red circles indicate where the ribonuclease activity was enriched.

(B) Fraction activity was examined with the in vitro degradation assay using biotinylated *UCP2* mRNA as a substrate.

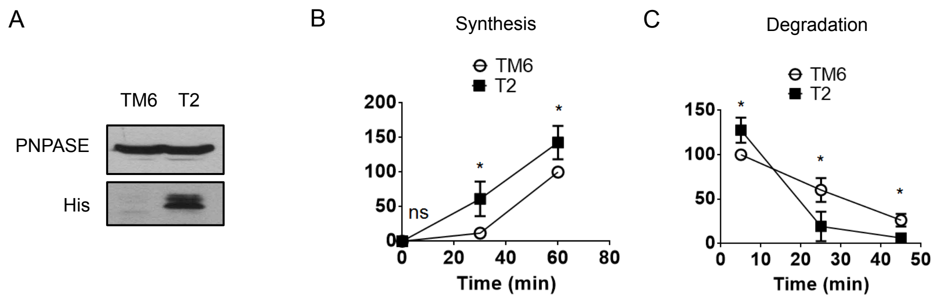

Figure S3. RNASET2 functions in mtRNA degradation in mouse cells.
(A) Immunoblots of mouse TM6 cell lysate (TM6) and RNASET2-overexpressing cell lysate (T2). PNPASE was used as a loading control.
(B) In organello mtRNA synthesis in control mitochondria (TM6) and mitochondria overexpressing RNASET2 (T2).
(C) In organello mtRNA degradation in control mitochondria (TM6) and mitochondria overexpressing RNASET2 (T2).
Statistical comparisons are performed using unpaired *t*-tests (n=3 if not specified); *P<0.05, **P<0.01, ***P<0.001, ****P<0.0001. Data are presented as mean ± standard error of the mean (s.e.m.).

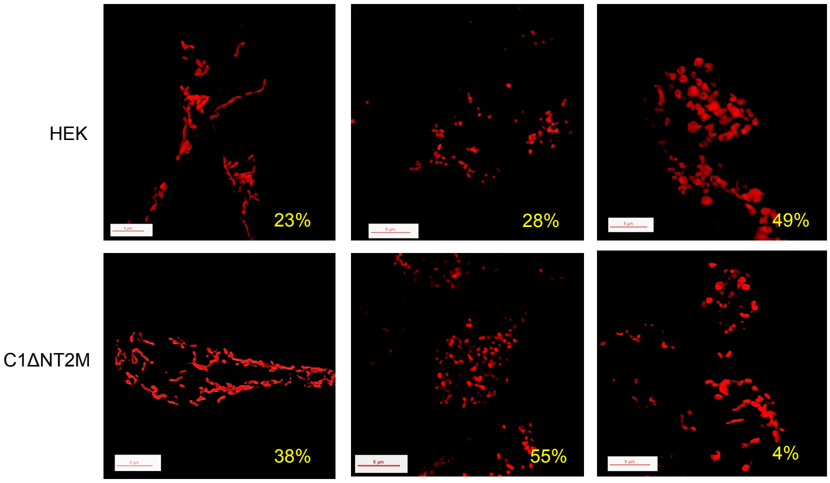

Figure S4. IMS-targeted RNASET2 dominant negative mutant (C1ΔNT2M) mitigates the effect of H_2O_2 treatment on mitochondrial structure. Fluorescent microscopy of HEK cells and C1ΔNT2M expressing cells. Yellow numbers represent the percentages of three populations with different mitochondrial structures. About 100 cells of each strain were counted.

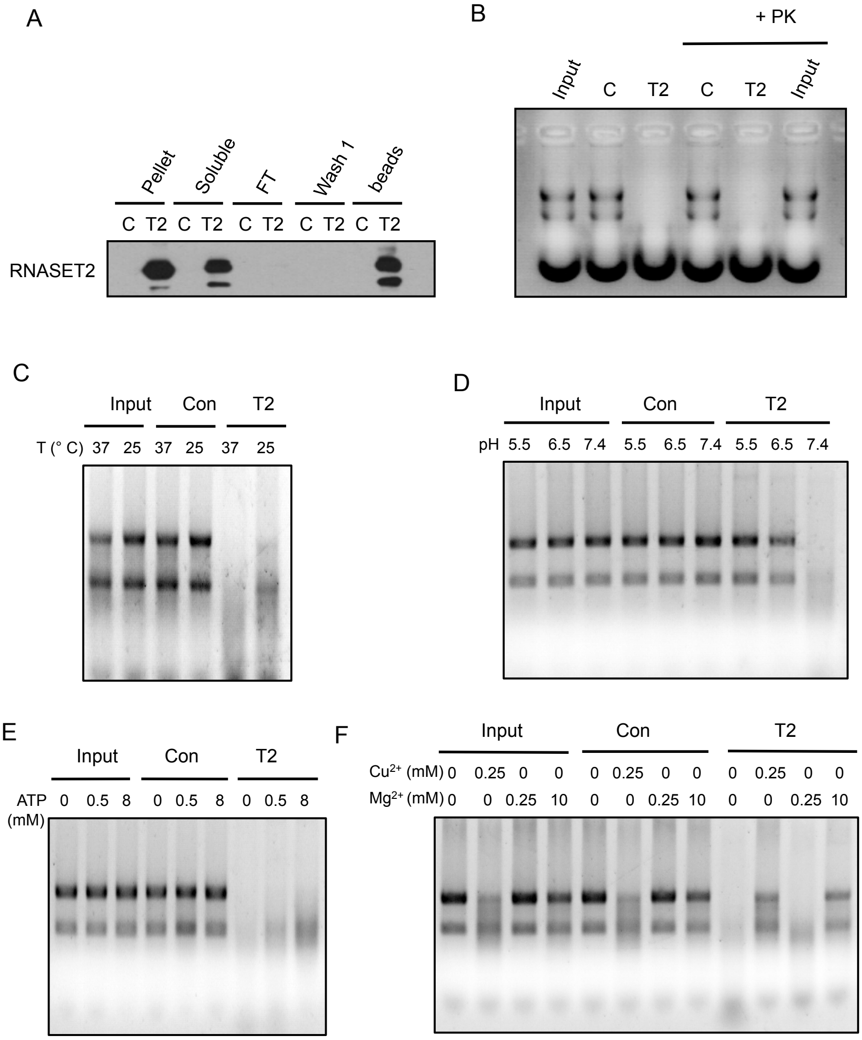

Figure S5. Characterization of human RNASET2 purified from bacteria.

(A) Purification of RNASET2 from bacteria under denaturing conditions, C (control pulldown from *E. coli* with empty vector) and T2 (RNASET2); FT (Flow through).

(B) RNASET2 purified from bacteria was checked for its ribonuclease activity and proteinase K sensitivity; C (control pulldown from *E. coli* with empty vector), T2 (RNASET2) and PK (Proteinase K).

(C) Effect of temperature on RNASET2 purified from bacteria.

(D) Effect of pH on RNASET2 purified from bacteria.

(E) Effect of ATP on RNASET2 purified from bacteria.

(F) Effect of Mg^{2+} and Cu^{2+} on RNASET2 purified from bacteria.

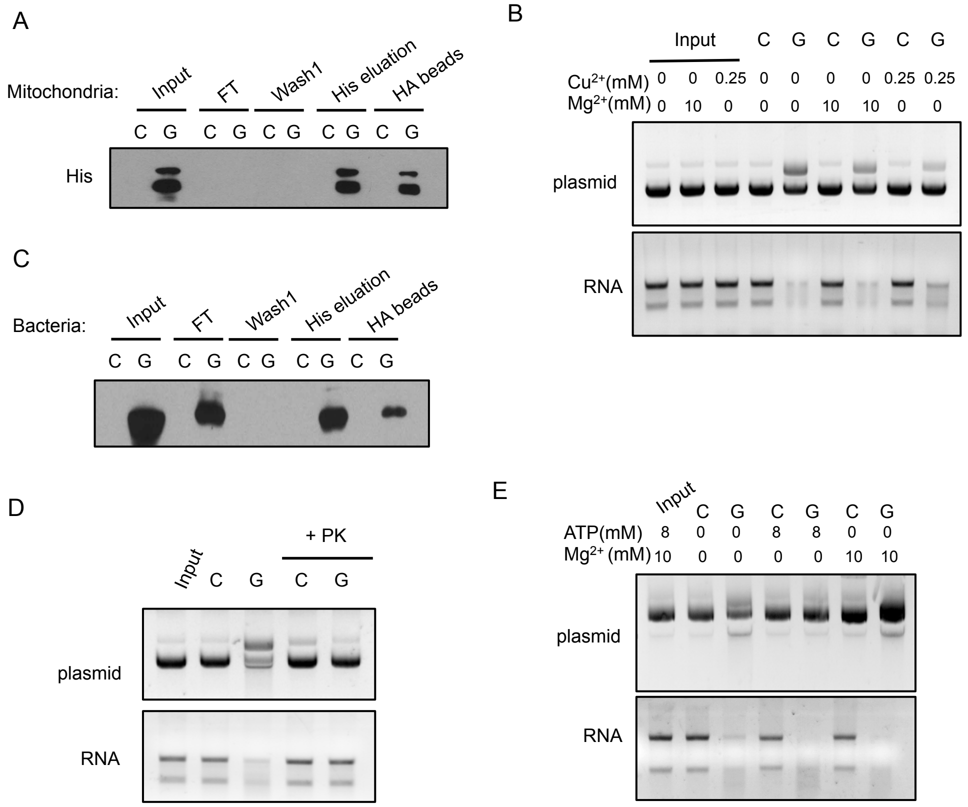

Figure S6. Endo G has different enzymatic characteristics from those of RNASET2. (A) Purification of duel tagged (His and HA) Endo G from HEK mitochondria, C (control pulldown from HEK mitochondria) and G (Endo G); FT (Flow through).

(B) Endo G purified from HEK mitochondria was checked for its DNase activity using PQCXIP plasmid as a substrate and ribonuclease activities using total cytosolic RNA as substrates. Different concentrations of Cu^{2+} and Mg^{2+} were used to check the sensitivity of the enzymatic activities to these metal ions; C (control pulldown from HEK mitochondria), and G (Endo G)

(C) Purification of duel tagged (His and HA) Endo G from *E. coli*, C (control pulldown from *E. coli* with empty vector) and G (Endo G); FT (Flow through).

(D) Effect of proteinase K treatment on the enzymatic activities of Endo G purified from E. coli; C (control pulldown from *E. coli* with empty vector) and G (Endo G)

(E) Effect of ATP or Mg^{2+} treatment on the enzymatic activities of Endo G purified from E. coli.

SUPPLEMENTAL EXPERIMENTAL PROCEDURES

Cell lines and culture

Hela, HEK293 and TM6 cells were cultured in DMEM supplemented with 10% fetal bovine serum. TM6 is a mouse mammary hyperplastic epithelial cell line. To generate stable cell lines, cells were co-transfected with the constructs of interest plus *VSVg* and *Hit60* packaging vectors using TurboFect (Thermo). Harvested retroviruses were used to infect HEK293 cells, followed by selection with 1 μg/ml puromycin. RNASET2 knockdown was achieved using shRNA-expressing constructs (Sigma-aldrich).

Plasmids

To construct *PQCXIP-RNASET2-HAHisPC* for mammalian transfection, *RNASET2* was PCR amplified from HEK293 cDNA library using the primers 5'-AACACCGGTATGCGCCCTGCAGCCCTG-3' and 5'-AAAGGATCCGGCGTAATCTGGAACATCGTATGGGTAACTTGTTCTATGCTTGGTCTTTT-3' and inserted into *PQCXIPHisPC* with AgeI and BamHI. To generate *PQCXIP-C1-ΔN-RNASET2-HAHisPC*, *ΔN-RNASET2* was PCR amplified with primers 5'-ACCGGTGATGgACAAGCGCCTG-3'and 5'-AAAGGATCCGGCGTAATCTGGAACATCGTATGGGTAACTTGTTCTATGCTTGGTCTTTT-3' and inserted into *PQCXIP-C1-HisPC* with AgeI and BamHI. To generate *PQCXIP-RNASET2-GFP*, *eGFP* was PCR amplified from PQSUPER with primers 5'-GGGATCCGGATGGTGAGCAAG-3' and 5'-GGAATTCTACACATTGATCCTAG-3' and inserted into *PQCXIP-RNASET2-HAHisPC* with BamHI and EcoRI. *RNASET2 C65R* and *C18R* site-directed mutagenesis was performed using the following primers: C65R sense, 5'-GGACAATATATGGACTATGGCCCGAT-3'; and C65R antisense, 5'-CCATATATTGTCCAGTAATCCGGAGG -3'; C184R sense, 5'-CCCATACTTTTCCCACTCATGC-3'; and C184R antisense, 5'-GGAAAAGTATGGGACCTGCGC-3'. To construct *PET28A-RNASET2-HisPC* for bacterial expression, *RNASET2* was PCR amplified with primers 5'-AACACCGGTATGCGCCCTGCAGCCCTG-3' and 5'-GAATTCCTAATGCTTGGTCTTTTTAGG-3' and inserted in to *PET28A* with AgeI and EcoRI.

APEX2 sequence was amplified from *pcDNA3 APEX2-NES* with primers 5’-CCGGATCCATGGACTACAAGGATGACG-3’ and 5’-CCGCTCGAGCTATTAGTCCAGGGTCAGG-3’. To construct *PQCXIP-APEX-HisPC*, the PCR product was digested with BamHI and XhoI and inserted into *PQCXIP-HisPC* vector. To generate *PQCXIP-MIA40-APEX-HisPC*, *Mia40* was amplified from HEK293 cDNA with primers 5’-ATTGCGGCCGCATGTCCTATTGCCGG-3’ and 5’-CGCGGATCCACTTGATCCCTCCTCTTC-3’, digested with NotI and BamHI, and inserted into *PQCXIP-APEX*. To construct *PQCXIP-RNASET2-APEX-HisPC*, *RNASET2* was digested from *PQCXIP-RNASET2-HAHis* with AgeI and XhoI and inserted into *PQCXIP-APEX*. To construct *PQCXIP-PNPASE-HAHis*, PNPASE was amplified with primers 5’-ATTTGCGGCCGCATGGCGGCCTGCAGGTAC-3’ and 5’-GTCGACCGGTTTAAGCGTAATCTGGAACATCGTATGGGTATCCTCCTGCTCCTCC-3’, and inserted into *PQCXIP-HAHis* with NotI and AgeI.

To construct *PQCXIP-MIA40-Avi-HisFlag*, MIA40 was amplified from *PQCXIP-MIA40-APEX-HisPC* with primers 5’-ATTGCGGCCGCATGTCCTATTGCCGG-3’ and 5’-CCCTCGAGTTCGTGCCATTCGATTTTCTGAGCCTCGAAGATGTCGTTC-3 that contains the Avi sequence, digested with NotI and XhoI, and inserted into *PQCXIP-HisFlag* vector. *PQCXIP-RNASET2-Avi-HisFlag* was constructed the same way with primers: 5’-AACACCGGTATGCGCCCTGCAGCCCTG-3’ and 5’-CCCTCGAGTTCGTGCCATTCGATTTTCTGAGCCTCGAAGATGTCGTTC-3’. BirA sequence was amplified from pBY2982-BirA-mCherry with primers: 5’-CGCGGATCCATGAAGGATAACACCGTGCC-3’ and 5’CCGCTCGAGTTTTTCTGCACTACGCAGGG-3’, digested with BamHI and XhoI, and inserted into *PQCXIP-HAHis* to generate *PQCXIP-BirA-HAHis*. TOM22 was amplied from HEK293 cDNA library with primers: 5’ATTTGCGGCCGCATGGCTGCCGCCGTCGCTG-3’ and 5’-CGCGGATCCGATCTTTCCAGAAGTGAGG-3’, digested with NotI and BamHI, and inserted into *PQCXIP-BirA-HisHA* to generate the final *PQCXIP-TOM22-BirA-HAHis.*

To construct *PQCXIP-EndoG-HAHis*, *EndoG* was PCR amplified from HEK293 cDNA with primers 5’-ATAACCGGTATGCGGGCGCTGCGGGCCG-3’ and 5’-CGCGGATCCCTTACTGCCCGCCGTGATGG-3’ and inserted into *PQCXIP-HAHis* with AgeI and BamHI. To construct *PET28A-EndoG-HisHA*, *EndoG* was PCR amplified

from HEK293 cDNA with primers 5'-ACTGGATCCATGCGGGCGCTGCGGGCCG-3' and 5'-CCGGAATTCCTTACTGCCCGCCGTGATGG-3' and inserted into *PET28A-HisHA* with BamHI and EcoRI.

Protein Purification

Purification of PNPASE and RNASET2 under native condition was performed as before (Claypool et al., 2008). Purification of RNASET2 under denature condition was performed using Ni-NTA beads (Qiagen) and Urea according to the supplier's instruction. Purification of Endo G was similar to that of PNPASE except the second purification was performed with HA beads (Thermo) using buffer D (20 mM Hepes, pH 7.4, 5% glycerol, 10 mM KCl, 1.5 mM $MgCl_2$, 1 mM EDTA, 1 mM EGTA and protease inhibitor cocktail).

Western Blotting

Cells were washed twice in 1xPBS, pH 7.4, and lysed in buffer A (10 mM HEPES, pH 7.9, 10 mM KCl, 1.5 mM $MgCl_2$ and 0.5% NP-40). Lysates were spun at 21 kg for 5 min to rid of nuclei. Mitochondria were lysed directly in 1X SDS loading buffer. Solubilized Mitochondrial fraction samples were prepared by adding 2X or 4X loading buffer depending on the concentration and the buffers used for solubilization. Protein lysates (50μg) were resolved by SDS-PAGE, transferred to nitrocellulose membranes, incubated for 1h with 5% milk TBS-T and overnight with primary antibodies in 5% BSA at 4 °C or for 1 to 2 h at room temperature. Antibodies included PNPASE (1:5000) (Rainey et al., 2006), LAMP1 (1:1000) (Sigma-Aldrich), HA monoclonal (1:1000) (Abclonal technology), His monoclonal (1:1000) (Abclonal technology), RNASET2 (1:1000) (Abgent), TIM23 (1:1000) (Abgent), Mortalin (1:10000) (Sigma-Aldrich).

Mitochondrial RNA isolation

100 - 200 μg mitochondria were heated at 90 °C in 100 μl lysate buffer (1% SDS, 10 mM Tris pH 7.4, and 10 mM EDTA) for 5 min, cooled to room temperature, treated with 1 μg of proteinase K at 37 °C for 5 min. 400 μl TRIzol (Invitrogen) was used for each 100 μl lysate. The RNA pellet was resuspended in 40 μl 1X DNase buffer with 0.5 μl DNase (Thermo), and incubated at 37 °C for 20 min with

one vortexing and centrifugation at 10 min. EDTA (5 mM from 50 mM stock) was then added and the sample was heated at 70 °C for 10 min to inactivate the DNase.

QPCR

100 of mitochondria from human cell lines were mixed with 100 μg of mouse TM6 mitochondria for mtRNA isolation. 2 μl of the final 45 μl RNA was used for a 20 μl cDNA synthesis reaction using the Superscript First Strand Synthesis System (Invitrogen) and specific reverse primers for mouse 12S rRNA, and human mitochondrial RNAs of interest. 0.3 μl of the 20 μl cDNA was used for a 10 μl SYBR green qPCR. Mouse 12S rRNA was used as control for the difference in human mitochondrial RNA levels. Primers used are 5'-TGGCTCAGCTGACTTACCAC-3' and 5'-TCTGGGTCTCCCCTCACAAT-3' for mouse 12S rRNA, 5'-TTTACACCCTCCTGAGCTCCT-3' and 5'-CATGGGGTTGGCACCATTGA-3' for human 12S rRNA, and 5'-TGCGGGGGCTTTGTATGATT-3' and 5'-CCTATTCCCCCGAGCAATCTC-3' for human ND6 mRNA.

Optiprep gradient centrifugation

Crude mitochondria were resuspend in 19% optiprep (Sigma, dilute with mitoprep buffer), layered on top of a 5 ml optiprep gradients (8%, 12%, 16%, 19%, 22.5%, and 27%), and centrifuged at 150 kg for 4 hours at 4 °C in a Beckman SW55-Ti rotor. 9 fractions were collected from the top, diluted with 1 ml mitoprep buffer and pelleted at 21 kg for 10 min.

Identification of IMS ribonuclease

Mitochondria Intermembrane Space (IMS) was subjected to molecular sieve chromatography and then mono Q chromatography on an AKTA explorer FPLC system (GE Healthcare) according to the column suppliers' instructions. After molecular sieve chromatography, the fractionated samples were tested for ribonuclease activity using an in vitro degradation assay. Fractions with ribonuclease activity were pooled together and run through mono Q chromatography. The fractionated samples were again tested for ribonuclease activity. Samples with activity were examined for proteinase K sensitivity.

Proteinase K resistant samples were concentrated, treated with proteinase K, and run on a 12% SDS-PAGE after proteinase K inactivation. The whole lanes were cut out, cut into three pieces, and sent for mass spectroscopy. Criterion for positive identification was identification of a minimum of 2 peptides with a Mascot peptide score ≥ 20. Final list was narrowed down by subtracting proteins identified in the control samples without activity (Table S1, 2).

Fluorescence microscopy and image acquisition

Hela cells transiently transfected with RNASET2-GFP expressing vector were grown in 35 mm glass plates to 70~80% confluency, stained with 100 nM Mitotracker red (invitrogen) for 30 min. Fluorescence images were taken with a Nikon-A1 confocal microscope.

In vitro transcription

RNAs were synthesized using MEGAscript SP6 Kit (Ambion) and purified with TRIzol reagent (Invitrogen). Biotin RNA labeling mix (Roche) was used to label the synthesized RNAs.

REFRENCES

Claypool, S.M., Oktay, Y., Boontheung, P., Loo, J.A., and Koehler, C.M. (2008). Cardiolipin defines the interactome of the major ADP/ATP carrier protein of the mitochondrial inner membrane. The Journal of cell biology *182*, 937-950.

Rainey, R.N., Glavin, J.D., Chen, H.W., French, S.W., Teitell, M.A., and Koehler, C.M. (2006). A new function in translocation for the mitochondrial i-AAA protease Yme1: import of polynucleotide phosphorylase into the intermembrane space. Molecular and cellular biology *26*, 8488-8497.

Table S1. Partial purification overlap proteome of fractions with ribonuclease activity (RNASET2 in red)

	Description	Score	Coverage	# Protein	ique Pep	# Peptide	# PSMs	# AAs	MW [kDa]	calc. pI
IPI00911039.1	cDNA FLJ54408, highly similar to He	1247.71	44.88	3	1	28	529	586	63.9	5.55
IPI00940148.1	Rab GDP dissociation inhibitor beta	1121.16	84.49	8	36	46	441	445	50.6	6.47
IPI00003865.1	Isoform 1 of Heat shock cognate 71	746.64	56.50	17	18	33	313	646	70.9	5.52
IPI00925850.2	cDNA FLJ54572, highly similar to Lys	617.12	48.00	3	34	34	272	950	107.4	7.02
IPI00022334.1	Ornithine aminotransferase, mitocho	585.74	59.45	2	31	31	217	439	48.5	7.03
IPI00843910.2	Tissue alpha-L-fucosidase	459.96	56.22	2	19	19	218	466	53.7	6.84
IPI00329633.5	Threonyl-tRNA synthetase, cytoplasn	437.41	63.07	10	45	49	174	723	83.4	6.67
IPI00007702.1	Heat shock-related 70 kDa protein 2	384.59	19.25	2	1	12	166	639	70.0	5.74
IPI00789370.3	serine hydroxymethyltransferase, mit	373.57	67.49	13	27	27	158	483	53.4	8.15
IPI00910113.1	cDNA FLJ52902, highly similar to Ral	342.11	30.70	3	2	13	169	417	46.9	5.05
IPI00219217.3	L-lactate dehydrogenase B chain	340.19	61.38	11	18	19	139	334	36.6	6.05
IPI00001593.1	Lysosomal Pro-X carboxypeptidase	336.96	32.06	12	16	16	138	496	55.8	7.21
IPI00012585.1	Beta-hexosaminidase subunit beta	313.90	49.28	6	27	27	133	556	63.1	6.76
IPI00011229.1	Cathepsin D	307.81	45.39	6	17	17	143	412	44.5	6.54
IPI00219005.3	Peptidyl-prolyl cis-trans isomerase Fk	289.95	40.31	2	22	22	108	459	51.8	5.43
IPI00012102.1	N-acetylglucosamine-6-sulfatase	281.20	43.66	8	21	21	143	552	62.0	8.31
IPI00019988.1	N-sulphoglucosamine sulphohydrolas	219.74	53.39	3	20	20	73	502	56.7	6.95
IPI00296141.4	Dipeptidyl peptidase 2	198.53	43.29	2	17	17	65	492	54.3	6.32
IPI00964764.1	cDNA FLJ55072, highly similar to Suc	184.71	47.73	9	2	23	80	616	67.3	8.12
IPI00965327.1	Uncharacterized protein	184.13	48.20	7	1	22	80	583	63.5	7.24
IPI00984405.1	Putative uncharacterized protein (Fra	179.57	35.45	11	10	16	71	457	51.7	9.03
IPI00169285.6	Putative phospholipase B-like 2	177.28	30.22	2	14	14	66	589	65.4	6.80
IPI00554786.5	Isoform 5 of Thioredoxin reductase 1	168.34	47.90	17	14	15	59	499	54.7	6.47
IPI00413451.3	Serpin B6	166.52	56.97	4	20	20	66	409	46.3	5.76
IPI01014578.1	Serine/threonine-protein phosphatas	161.70	37.60	6	16	16	68	484	55.1	6.20
IPI00967269.1	Uncharacterized protein	159.55	48.82	8	12	12	55	254	29.2	6.76
IPI00001960.4	Chloride intracellular channel protein	155.13	69.17	11	15	15	59	253	28.8	5.59
IPI00186290.6	Elongation factor 2	154.13	25.64	6	19	19	58	858	95.3	6.83
IPI00217966.9	Isoform 1 of L-lactate dehydrogenase	152.21	64.16	25	19	20	75	332	36.7	8.27
IPI00472165.1	Isoform 1 of Procollagen-lysine,2-oxo	147.55	33.65	6	22	22	65	737	84.6	6.71
IPI00479018.3	Isoform 2 of Syntenin-1	147.01	47.81	7	8	8	49	297	32.3	7.53
IPI00027745.4	Isoform Long of Beta-glucuronidase	142.77	27.50	9	16	16	53	651	74.7	7.02
IPI00012007.6	Adenosylhomocysteinase	140.82	39.81	11	18	18	71	432	47.7	6.34
IPI00011201.1	NAD-dependent malic enzyme, mitoc	133.84	52.57	2	22	22	56	584	65.4	7.61
IPI00549189.4	Thimet oligopeptidase	126.14	42.82	2	27	27	56	689	78.8	6.05
IPI00013860.3	3-hydroxyisobutyrate dehydrogenase	125.25	45.24	2	13	13	46	336	35.3	8.13
IPI00293655.3	ATP-dependent RNA helicase DDX1	124.98	25.14	3	18	18	48	740	82.4	7.23
IPI00010346.1	Neurolysin, mitochondrial	120.37	43.47	6	30	30	51	704	80.6	6.64
IPI00029997.1	6-phosphogluconolactonase	119.55	67.05	1	15	15	50	258	27.5	6.05
IPI00291783.4	Gem-associated protein 5	119.08	18.30	1	24	24	62	1508	168.5	6.62
IPI00293088.7	Lysosomal alpha-glucosidase	113.60	31.51	1	14	22	50	952	105.3	6.00
IPI00218728.4	Isoform 1 of Platelet-activating facto	112.41	44.39	5	14	14	53	410	46.6	7.37
IPI00446007.3	cDNA FLJ39087 fis, clone NT2RP701	105.83	34.73	5	10	10	29	357	40.2	7.77
IPI01011421.2	Uncharacterized protein	105.81	33.33	7	14	14	42	459	48.9	7.49
IPI00375426.8	Uncharacterized protein	105.47	38.08	3	9	9	47	323	36.2	8.10
IPI01012741.1	cDNA FLJ51866, highly similar to Trif	100.34	27.40	4	10	10	36	562	60.2	6.76
IPI00013698.3	N-acylsphingosine amidohydrolase (A	99.88	22.71	4	16	16	51	546	60.6	8.81
IPI00029629.4	E3 ubiquitin/ISG15 ligase TRIM25	98.60	30.48	1	17	17	39	630	70.9	8.09
IPI00257882.7	Xaa-Pro dipeptidase	87.83	32.66	3	10	10	29	493	54.5	6.00
IPI01014382.2	Uncharacterized protein	86.17	47.14	8	18	18	40	490	54.3	7.80

IPI00472887.3	Isoform 2 of Cytoskeleton-associatec	83.60	11.41	4	19	19	37	1972	218.4	8.06
IPI00026260.1	Isoform 1 of Nucleoside diphosphate	78.60	59.21	9	4	8	37	152	17.3	8.41
IPI01015565.1	Uncharacterized protein	77.90	63.09	25	4	4	35	149	16.8	6.58
IPI00465186.3	Isoform 2 of Receptor-type tyrosine-|	72.79	14.54	17	17	17	35	1898	211.6	6.42
IPI00026259.2	N(4)-(beta-N-acetylglucosaminyl)-L-a	72.08	37.57	4	9	9	36	346	37.2	6.28
IPI00465436.4	Catalase	69.19	33.40	1	14	14	30	527	59.7	7.39
IPI00924935.1	cDNA FLJ57106, highly similar to Tra	68.69	30.63	3	18	18	31	679	75.9	6.81
IPI00029605.1	N-acetylgalactosamine-6-sulfatase	68.60	32.57	2	14	14	24	522	58.0	6.74
IPI00220342.5	N(G),N(G)-dimethylarginine dimethyl	67.87	48.42	4	10	10	21	285	31.1	5.81
IPI00328243.2	Phospholipase D3	67.86	21.63	3	8	8	23	490	54.7	6.47
IPI00644576.1	Filamin A, alpha	67.85	19.18	10	33	33	44	2607	276.4	6.05
IPI01012178.1	Ceroid-lipofuscinosis neuronal protei	66.21	26.26	3	9	9	31	358	41.5	7.40
IPI00012048.1	Isoform 1 of Nucleoside diphosphate	63.61	40.13	5	1	5	33	152	17.1	6.19
IPI00892711.1	Isoform 2 of Probable threonyl-tRNA	62.86	9.34	3	2	6	17	707	81.5	6.24
IPI01010585.1	cDNA FLJ58994, highly similar to Col	60.31	17.57	5	6	6	26	404	43.6	6.89
IPI00022314.1	Superoxide dismutase [Mn], mitocho	58.82	55.41	10	9	9	25	222	24.7	8.25
IPI00921849.1	cDNA FLJ57046, highly similar to Lys	58.34	18.63	1	3	11	22	644	71.7	6.34
IPI00301459.2	Group XV phospholipase A2	57.55	27.18	4	8	8	26	412	46.6	6.73
IPI00003815.3	Rho GDP-dissociation inhibitor 1	56.30	30.39	3	7	7	28	204	23.2	5.11
IPI00006663.1	Aldehyde dehydrogenase, mitochond	55.78	31.33	2	14	14	28	517	56.3	7.05
IPI00246058.10	Programmed cell death 6-interacting	54.25	33.87	6	23	23	30	868	96.0	6.52
IPI01014863.1	Acetyl-CoA acetyltransferase, cytosol	53.88	23.17	2	7	7	20	397	41.3	6.92
IPI00844513.1	Similar to Chain , Heat-Shock Cognat	52.73	21.21	1	1	5	22	231	25.6	7.61
IPI00010949.3	Isoform 1 of Sialate O-acetylesterase	52.47	26.20	2	12	12	30	523	58.3	7.33
IPI00027438.2	Flotillin-1	51.00	37.70	19	12	12	22	427	47.3	7.49
IPI00021997.1	Protein CREG1	50.40	27.73	1	4	4	16	220	24.1	7.59
IPI00943894.1	glycogen phosphorylase, liver form is	49.89	23.74	6	15	18	26	813	93.1	7.30
IPI00552937.1	NHL repeat-containing protein 3	49.23	29.97	4	9	9	18	347	38.3	6.43
IPI00241860.4	Mitochondrial intermediate peptidase	48.84	27.63	1	17	17	27	713	80.6	7.05
IPI00375688.1	Isoform 2 of Nicastrin	48.20	22.21	8	9	9	15	689	76.7	5.68
IPI00030320.4	Probable ATP-dependent RNA helicas	46.49	20.08	1	10	10	31	483	54.4	8.66
IPI00179964.5	Isoform 1 of Polypyrimidine tract-bin	45.38	17.70	5	7	7	16	531	57.2	9.17
IPI00013452.11	Bifunctional aminoacyl-tRNA syntheta	45.23	10.12	1	11	11	22	1512	170.5	7.33
IPI00787827.1	Isoform 2 of Presequence protease,	44.41	25.43	9	18	18	21	1038	117.5	6.98
IPI00927191.2	Isoform 3 of Beta-galactosidase	43.75	23.34	8	11	11	22	647	72.7	6.25
IPI00015856.6	aspartyl aminopeptidase	43.63	25.98	12	10	10	26	485	53.4	7.58
IPI00903145.1	Radixin	42.95	16.98	9	2	11	24	583	68.5	6.37
IPI00305978.4	Aflatoxin B1 aldehyde reductase mer	42.26	27.58	8	6	6	20	359	39.6	7.17
IPI00746165.2	Isoform 1 of WD repeat-containing p	41.75	39.77	7	14	14	22	606	66.2	6.65
IPI00216691.5	Profilin-1	41.24	59.29	1	7	7	21	140	15.0	8.27
IPI00220766.5	Lactoylglutathione lyase	40.40	40.76	1	8	8	17	184	20.8	5.31
IPI00843975.1	Ezrin	40.39	14.51	6	1	10	23	586	69.4	6.27
IPI00413958.5	Isoform 2 of Filamin-C	40.18	9.77	3	15	17	21	2692	287.1	5.97
IPI00178767.1	Acid sphingomyelinase-like phosphoc	40.08	18.76	2	7	7	13	453	51.2	6.33
IPI00646689.1	Thioredoxin domain-containing prote	39.11	54.47	1	5	5	21	123	13.9	5.52
IPI00021828.1	Cystatin-B	38.01	45.92	1	3	3	21	98	11.1	7.56
IPI00397768.5	Isoform 2 of Ribonucleoprotein PTB-	37.82	9.44	5	6	6	16	678	72.9	7.47
IPI00465044.2	Protein RCC2	37.51	36.78	1	15	15	23	522	56.0	8.78
IPI01014238.1	cDNA FLJ53963, highly similar to Leu	37.13	34.60	6	11	11	17	341	38.7	6.67
IPI00984060.1	glutathione S-transferase Mu 2 isofor	35.63	43.98	14	3	8	19	191	22.6	5.31
IPI00009844.1	Isoform 1 of GMP reductase 2	34.10	19.54	6	5	5	18	348	37.9	7.23
IPI00217920.7	Isoform 1 of Aldehyde dehydrogenas	32.86	13.22	5	8	8	17	802	85.1	6.79

IPI00604401.2	Isoform 2 of Epididymis-specific alph	32.60	14.47	3	11	11	14	954	107.6	7.05
IPI00018236.2	Ganglioside GM2 activator	31.80	43.52	4	6	6	14	193	20.8	5.31
IPI00007778.1	Di-N-acetylchitobiase	31.56	25.71	2	6	6	9	385	43.7	6.64
IPI01009249.1	Uncharacterized protein	31.24	16.12	2	6	6	15	490	54.8	7.50
IPI00940377.1	Uncharacterized protein	31.18	32.40	11	1	6	15	179	21.1	8.32
IPI00021327.3	Isoform 1 of Growth factor receptor-	31.04	20.74	2	5	5	12	217	25.2	6.32
IPI00004358.4	Glycogen phosphorylase, brain form	30.25	16.01	6	8	11	13	843	96.6	6.86
IPI00246975.8	Glutathione S-transferase Mu 3	30.23	32.89	2	5	6	20	225	26.5	5.54
IPI00884105.2	Lysosome-associated membrane glyc	29.93	12.95	3	6	6	12	417	44.9	8.75
IPI00646304.4	Peptidyl-prolyl cis-trans isomerase B	29.86	34.72	1	7	7	18	216	23.7	9.41
IPI00789396.2	Methionine aminopeptidase	29.43	16.04	3	7	7	15	455	50.5	5.92
IPI00069309.6	Leucine-rich repeat and WD repeat-c	27.36	16.38	3	7	7	14	647	70.8	7.21
IPI00014808.1	Platelet-activating factor acetylhydrol	27.12	22.94	1	4	4	14	231	25.7	6.84
IPI01010466.1	Uncharacterized protein	25.44	11.32	2	6	6	13	583	66.9	8.15
IPI00922369.1	3-oxoacyl-[acyl-carrier-protein] synth	25.35	28.19	5	6	6	10	376	40.0	7.88
IPI00306576.1	Arylsulfatase B	25.03	15.01	2	5	5	9	533	59.6	8.21
IPI00657648.1	mammalian ependymin-related prote	24.68	11.63	4	4	4	11	344	38.1	9.58
IPI00893035.1	Putative uncharacterized protein CAD	24.68	3.38	4	5	5	10	2162	235.9	6.55
IPI00797148.1	Isoform 2 of Heterogeneous nuclear	24.68	38.58	9	6	7	9	267	29.4	9.14
IPI00218465.10	Phospholipase A-2-activating protein	24.42	16.60	5	9	9	13	795	87.1	6.37
IPI00900380.1	Isoform 2 of Malignant T cell-amplifie	23.98	46.75	3	6	6	9	169	19.2	8.25
IPI01013799.1	Uncharacterized protein	23.30	11.81	5	4	4	8	542	59.4	6.16
IPI00922554.1	cDNA FLJ51996, highly similar to De	23.21	14.10	2	4	4	11	305	33.6	8.32
IPI00867514.3	Uncharacterized protein	22.63	24.47	4	5	5	14	282	32.0	6.96
IPI00909303.3	cDNA FLJ58073, moderately similar t	22.60	20.88	15	5	5	13	273	30.7	6.62
IPI00853369.1	Plexin-B2	22.28	10.77	4	13	13	14	1838	205.0	6.24
IPI00017672.4	cDNA FLJ25678 fis, clone TST04067,	22.25	27.30	1	6	6	9	293	32.5	7.21
IPI00005794.2	Uncharacterized protein	22.19	15.56	7	6	6	13	540	59.7	7.93
IPI00386427.1	Isoform 2 of Acid sphingomyelinase-l	22.12	15.28	4	4	4	9	373	41.7	6.24
IPI00929290.1	Isoform 2 of Protein argonaute-2	22.09	18.06	4	7	11	13	825	93.6	9.26
IPI00218493.7	Hypoxanthine-guanine phosphoribos	21.86	37.61	4	7	7	13	218	24.6	6.68
IPI00879937.1	Uncharacterized protein	20.80	4.31	3	1	1	12	255	27.8	6.77
IPI00939169.1	Isoform 3 of Attractin	20.77	7.85	3	8	8	11	1198	133.6	6.98
IPI00016342.1	Ras-related protein Rab-7a	20.00	34.78	7	6	6	7	207	23.5	6.70
IPI00927101.1	Uncharacterized protein	20.00	20.08	9	6	6	9	264	29.5	5.25
IPI00978762.1	voltage-gated potassium channel sub	19.98	14.00	13	5	5	10	300	33.6	8.35
IPI01012490.1	cDNA FLJ56675, highly similar to Act	19.53	21.18	2	4	4	9	288	32.3	5.72
IPI00980612.1	dynamin-2 isoform 5	18.45	7.13	13	6	6	12	869	97.9	7.44
IPI00883598.3	Uncharacterized protein	18.30	23.89	8	6	7	10	494	53.5	6.52
IPI00024403.1	Copine-3	17.74	16.20	20	7	7	13	537	60.1	5.85
IPI00867663.1	Isoform 2 of L-asparaginase	17.67	37.78	3	5	5	9	180	19.0	7.88
IPI00183065.6	Uncharacterized protein	17.40	11.34	4	5	5	7	573	63.8	9.88
IPI00006114.5	Pigment epithelium-derived factor	17.15	12.68	1	4	4	7	418	46.3	6.38
IPI00219677.4	Putative deoxyribose-phosphate aldo	17.10	27.67	8	7	7	8	318	35.2	8.94
IPI00794777.1	Uncharacterized protein	17.09	22.79	4	4	4	5	136	15.1	6.13
IPI00908781.2	thioredoxin reductase 3 isoform 2	16.94	16.47	2	7	7	8	607	66.5	8.28
IPI01010848.2	Uncharacterized protein	16.82	23.51	4	5	5	7	336	37.8	6.90
IPI00945027.1	Uncharacterized protein	16.39	7.13	11	3	3	4	547	61.6	7.53
IPI01015222.1	cDNA FLJ54259, highly similar to Sm	16.28	34.94	2	7	7	11	352	39.3	8.25
IPI00789806.2	Isoform 2 of Cytosol aminopeptidase	16.17	18.85	5	7	7	12	488	52.7	6.74
IPI00982620.2	cDNA FLJ61765, highly similar to 4-t	16.04	14.86	2	5	5	10	424	46.4	5.54
IPI00794805.1	Uncharacterized protein	16.02	18.87	4	2	2	7	159	18.3	9.48

IPI00964648.1	30 kDa protein	15.93	21.46	17	5	6	11	261	29.7	9.16
IPI00945881.1	Uncharacterized protein	15.75	35.86	2	1	7	10	237	26.1	8.90
IPI00027310.5	Isoform 1 of Multiple epidermal grow	15.62	4.75	2	8	8	9	2845	302.9	6.87
IPI00007736.5	Protein argonaute-1	15.45	14.24	3	6	9	10	857	97.2	9.16
IPI00009901.1	Nuclear transport factor 2	15.37	69.29	5	5	5	6	127	14.5	5.38
IPI00985156.1	Uncharacterized protein	15.36	23.53	12	4	4	5	323	34.5	8.59
IPI00007249.3	Ectonucleotide pyrophosphatase/pho	15.28	9.49	2	4	4	5	453	51.6	6.15
IPI01014515.1	Uncharacterized protein	14.77	31.49	8	8	8	10	289	33.8	5.45
IPI00298793.4	Beta-mannosidase	14.45	10.01	2	7	7	7	879	100.8	5.52
IPI00399089.4	LDLR chaperone MESD	14.38	13.25	1	3	3	6	234	26.1	7.78
IPI00018196.6	Isoform 1 of Notchless protein homo	14.04	20.62	5	7	7	8	485	53.3	7.34
IPI00940548.1	Oxysterol-binding protein	14.04	20.88	3	5	5	10	407	45.6	7.17
IPI00945725.1	cDNA FLJ58035, highly similar to Ho	13.82	18.57	4	2	8	11	560	62.2	8.91
IPI01013044.2	Uncharacterized protein	13.79	5.13	5	2	2	8	585	66.3	6.77
IPI00925601.1	Uncharacterized protein	13.77	11.68	6	6	6	11	591	64.5	6.71
IPI00384548.2	Lysosome-associated membrane prot	13.71	15.81	3	4	4	8	215	23.4	7.84
IPI01010588.1	cDNA FLJ54237, highly similar to Nu	13.52	13.76	4	2	2	7	218	23.6	5.76
IPI00878818.1	Isoform 3 of Aldehyde dehydrogenas	13.49	13.76	9	6	6	7	516	57.2	6.32
IPI00796213.2	Putative uncharacterized protein	13.40	17.50	8	3	4	6	320	35.5	8.78
IPI00647400.1	Uncharacterized protein	13.26	5.56	2	4	4	8	611	68.1	6.01
IPI00791053.2	aminoacylase-1 isoform b	13.18	26.79	8	6	6	9	336	37.6	5.91
IPI00515016.1	NAD(P)H dehydrogenase, quinone 2	13.00	29.02	4	3	3	6	193	21.5	6.52
IPI00383539.5	Citrate synthase	12.52	10.82	3	4	4	7	453	50.4	7.90
IPI00376503.2	pyrroline-5-carboxylate reductase 1,	12.25	11.39	8	3	3	6	316	33.3	6.80
IPI01012902.2	cDNA FLJ60958, highly similar to Ho	12.06	16.61	3	5	5	6	277	31.7	5.50
IPI00983581.1	SYNCRIP protein (Fragment)	12.05	17.66	7	5	7	9	453	50.6	6.71
IPI01015329.1	cDNA FLJ52203, highly similar to 3'(	11.80	20.55	6	3	3	6	253	27.5	5.33
IPI00398758.1	Isoform 2 of Enoyl-CoA delta isomera	11.76	10.88	2	3	3	5	285	30.9	8.90
IPI00005107.2	Niemann-Pick C1 protein	11.69	4.69	3	4	4	4	1278	142.1	5.36
IPI00025347.4	Ribosomal RNA small subunit methyl	11.64	19.67	1	3	3	5	244	26.7	9.17
IPI01011344.1	Uncharacterized protein	11.60	13.51	39	3	3	5	333	37.4	5.58
IPI00029744.1	Single-stranded DNA-binding protein	11.52	12.84	1	2	2	3	148	17.2	9.60
IPI00374151.1	thioredoxin-dependent peroxide redu	11.51	15.55	2	3	3	5	238	25.8	7.46
IPI00791474.1	Uncharacterized protein	11.20	25.50	7	4	4	7	149	17.5	9.19
IPI00479304.1	Isoform 3 of Contactin-1	11.07	9.73	3	4	4	7	627	70.6	6.11
IPI00026546.1	Platelet-activating factor acetylhydrol	10.67	17.47	5	3	3	4	229	25.6	5.92
IPI00304181.4	Isoform 1 of Protein argonaute-3	10.32	8.37	3	1	5	6	860	97.3	9.11
IPI00977736.1	calcyclin-binding protein isoform 2	9.96	36.76	3	4	4	5	185	21.2	7.90
IPI00965290.1	Isoform 3 of Cullin-4B	9.79	5.02	4	3	4	4	717	84.0	6.79
IPI00646292.1	20 kDa protein	9.77	28.89	25	3	3	3	180	20.4	6.86
IPI00219617.5	Isoform 1 of Ribose-phosphate pyrop	9.48	15.41	11	3	3	4	318	34.7	6.61
IPI00060031.3	ADP-ribosylation factor-like protein 8	9.48	29.57	3	3	3	3	186	21.4	7.77
IPI00003927.5	Peptidyl-prolyl cis-trans isomerase D	9.47	14.32	1	4	4	4	370	40.7	7.21
IPI00981773.1	cDNA FLJ53377, highly similar to Pro	9.37	10.14	5	5	5	5	690	79.3	7.02
IPI00023549.5	Galectin-related protein	9.21	19.77	1	3	3	6	172	19.0	5.35
IPI00008215.1	NADP-dependent malic enzyme	9.18	12.59	4	5	5	6	572	64.1	6.13
IPI00305010.4	Isoform 1 of Calcineurin-like phosph	9.15	14.33	3	4	4	5	314	35.5	6.20
IPI00902914.2	Uncharacterized protein	9.10	7.15	2	4	4	5	629	71.5	7.56
IPI00790702.1	Beta adaptin subunit	8.98	11.36	7	7	8	10	880	98.1	5.24
IPI00016670.3	Ragulator complex protein LAMTOR1	8.94	23.60	5	3	3	5	161	17.7	5.15
IPI00412880.2	Isoform 1 of Histone-arginine methyl	8.91	8.21	3	4	4	5	585	63.4	6.57
IPI00008164.2	Prolyl endopeptidase	8.77	10.28	1	5	5	6	710	80.6	5.86

IPI01010611.1	Uncharacterized protein	8.71	12.71	7	3	3	4	236	26.6	5.38
IPI00025869.1	Alpha-galactosidase A	8.64	13.05	1	4	4	6	429	48.7	5.60
IPI00640741.1	19 kDa protein	8.57	18.71	6	3	3	5	171	19.0	6.92
IPI01014903.2	Uncharacterized protein	8.49	3.04	2	2	2	2	1085	124.5	8.60
IPI00953696.1	glutathione reductase, mitochondrial	8.47	11.59	5	3	3	4	440	47.2	8.75
IPI00946636.2	cDNA FLJ51804, highly similar to Vac	8.42	11.13	4	5	5	6	584	64.7	5.66
IPI00908636.1	cDNA FLJ61130	8.20	13.62	6	3	3	3	323	34.7	8.82
IPI00003527.5	Na(+)/H(+) exchange regulatory cof	8.17	14.25	2	5	5	6	358	38.8	5.77
IPI00005492.2	WD repeat-containing protein 5	8.15	12.28	1	3	3	4	334	36.6	8.27
IPI00845372.1	Isoform 2 of Fucose mutarotase	8.00	34.33	3	3	3	3	134	14.6	5.26
IPI00877061.1	Isoform 3 of Protein tweety homolog	7.94	8.24	3	2	2	5	352	38.5	6.34
IPI00910614.1	heterogeneous nuclear ribonucleopro	7.88	8.27	5	2	4	5	532	59.6	9.16
IPI00218236.6	Serine/threonine-protein phosphatas	7.80	15.29	14	4	4	5	327	37.2	6.19
IPI01012499.1	Uncharacterized protein	7.68	8.96	12	3	3	4	268	30.3	8.35
IPI01015174.2	Uncharacterized protein	7.63	7.94	4	3	3	3	592	66.4	7.91
IPI00103732.1	Thymidylate synthetase, isoform CRA	7.58	12.19	3	3	3	4	279	31.7	7.01
IPI00012493.1	40S ribosomal protein S20	7.53	19.33	5	2	2	3	119	13.4	9.94
IPI00020906.1	Inositol monophosphatase 1	7.48	23.10	11	5	5	6	277	30.2	5.26
IPI00551062.2	Isoform 1 of Protein canopy homolog	7.43	6.47	1	1	1	3	278	30.7	5.49
IPI00953109.2	Isoform 6 of Filamin-B	7.42	2.21	9	2	4	4	2537	271.2	5.72
IPI01013355.2	Uncharacterized protein	7.22	9.40	4	5	5	6	702	77.9	6.95
IPI00026530.4	Protein ERGIC-53	7.14	15.69	1	3	3	4	510	57.5	6.77
IPI00465022.9	Isoform 2 of Structural maintenance	7.11	3.29	3	5	5	6	1917	215.6	7.01
IPI00607548.4	Isoform 5 of InaD-like protein	7.04	0.53	7	1	1	5	1134	125.2	4.88
IPI00979862.1	Uncharacterized protein	6.87	16.20	7	2	2	3	179	20.3	8.68
IPI00220362.5	10 kDa heat shock protein, mitochon	6.81	25.49	5	2	2	3	102	10.9	8.92
IPI01011948.1	glucosylceramidase isoform 2	6.78	6.01	14	2	2	3	449	50.3	7.15
IPI00549919.1	Isoform 2 of Cullin-4A	6.77	3.95	2	1	2	2	659	76.8	7.18
IPI00914938.1	Isoform 1 of AP-2 complex subunit a	6.72	5.54	6	3	4	6	939	103.9	6.96
IPI00909703.2	Uncharacterized protein	6.71	12.14	4	4	4	5	412	45.7	8.46
IPI00924676.1	Putative uncharacterized protein GTF	6.70	17.52	8	4	4	4	274	30.4	5.01
IPI00964913.1	Uncharacterized protein	6.69	11.15	5	3	3	3	260	29.3	4.94
IPI00967230.1	Uncharacterized protein	6.59	9.87	4	3	3	3	395	44.9	8.62
IPI00984414.2	Elongation factor 1-alpha	6.59	5.62	8	2	2	5	427	46.4	7.44
IPI00554617.2	cDNA FLJ57277, highly similar to Trip	6.47	7.27	4	2	2	4	385	41.6	5.45
IPI00644458.1	SM-11044 binding protein	6.44	12.20	2	3	3	4	254	29.9	5.82
IPI00412404.1	ATP-dependent RNA helicase SUPV3I	6.44	7.51	1	4	4	5	786	87.9	7.99
IPI00216298.6	Thioredoxin	6.43	23.81	2	2	2	8	105	11.7	4.92
IPI00853224.1	StAR-related lipid transfer protein 7,	6.40	7.57	2	2	2	4	370	43.1	8.95
IPI00181911.4	Isoform 2 of Tetratricopeptide repeat	6.32	6.51	3	3	3	4	522	59.2	7.30
IPI00298281.4	Laminin subunit gamma-1	6.29	3.73	1	4	4	4	1609	177.5	5.12
IPI00004669.1	Polypeptide N-acetylgalactosaminyltr	6.28	10.33	4	4	4	4	571	64.7	8.35
IPI00872761.2	fragile X mental retardation 1 protein	6.22	12.21	12	5	5	5	516	58.8	6.32
IPI00795504.3	Uncharacterized protein	6.17	4.70	5	2	2	4	532	59.5	6.10
IPI00925025.1	Uncharacterized protein	6.12	19.13	7	1	3	3	230	25.4	6.80
IPI00217766.3	Lysosome membrane protein 2	6.06	6.07	3	2	2	2	478	54.3	5.14
IPI00176427.1	Cell adhesion molecule 4	5.94	6.44	1	2	2	2	388	42.8	6.30
IPI00641992.4	Adenylosuccinate synthetase	5.94	15.40	2	5	5	9	435	47.9	5.91
IPI00215844.1	Isoform 2 of N-acylethanolamine-hyd	5.87	9.60	6	3	3	4	323	35.9	7.53
IPI00220578.3	Guanine nucleotide-binding protein G	5.82	10.73	31	3	3	5	354	40.5	5.69
IPI00917207.1	Uncharacterized protein	5.62	17.37	9	3	3	3	213	24.6	7.53
IPI00384489.2	AP-1 complex subunit beta-1 isoform	5.44	4.13	6	2	3	4	919	101.3	5.11

IPI00924593.1	cDNA FLJ52880, highly similar to Ma	5.28	6.42	2	1	1	1	296	30.9	8.43
IPI00914848.1	Isoform 2 of Glia-derived nexin	5.25	10.08	4	3	3	3	397	44.0	9.38
IPI00747807.1	Isoform 3 of Endoplasmic reticulum a	5.20	4.04	7	3	3	3	915	105.5	6.84
IPI00644108.3	32 kDa protein	5.18	16.19	5	4	4	6	278	31.7	8.16
IPI00411579.3	Uncharacterized protein	5.09	8.96	6	2	2	2	357	41.1	7.47
IPI00063902.4	Uncharacterized protein	5.08	8.14	4	2	2	2	258	29.1	8.03
IPI01011409.1	zinc phosphodiesterase ELAC protein	5.00	5.94	5	4	4	4	825	92.0	7.81
IPI00646055.3	Uncharacterized protein	5.00	5.38	2	2	3	3	651	74.2	7.87

Table S2. Ribonuclease activity-positive proteome substrated by activity-negative proteome

	Description	Score	Coverage	Protei	que Pe	Peptide	# PSMs	# AAs	MW [kDa]	calc. pI
IPI00925850.2	cDNA FLJ54572, highly similar to Lysosom	617.12	48.00	3	34	34	272	950	107.4	7.02
IPI00843910.2	Tissue alpha-L-fucosidase	459.96	56.22	2	19	19	218	466	53.7	6.84
IPI00329633.5	Threonyl-tRNA synthetase, cytoplasmic	437.41	63.07	10	45	49	174	723	83.4	6.67
IPI00007702.1	Heat shock-related 70 kDa protein 2	384.59	19.25	2	1	12	166	639	70.0	5.74
IPI00011229.1	Cathepsin D	307.81	45.39	6	17	17	143	412	44.5	6.54
IPI00012102.1	N-acetylglucosamine-6-sulfatase	281.20	43.66	8	21	21	143	552	62.0	8.31
IPI00219005.3	Peptidyl-prolyl cis-trans isomerase FKBP4	289.95	40.31	2	22	22	108	459	51.8	5.43
IPI00019988.1	N-sulphoglucosamine sulphohydrolase	219.74	53.39	3	20	20	73	502	56.7	6.95
IPI00984405.1	Putative uncharacterized protein (Fragmen	179.57	35.45	11	10	16	71	457	51.7	9.03
IPI00012007.6	Adenosylhomocysteinase	140.82	39.81	11	18	18	71	432	47.7	6.34
IPI01014578.1	Serine/threonine-protein phosphatase (Fra	161.70	37.60	6	16	16	68	484	55.1	6.20
IPI00472165.1	Isoform 1 of Procollagen-lysine,2-oxogluta	147.55	33.65	6	22	22	65	737	84.6	6.71
IPI00291783.4	Gem-associated protein 5	119.08	18.30	1	24	24	62	1508	168.5	6.62
IPI00554786.5	Isoform 5 of Thioredoxin reductase 1, cyto	168.34	47.90	17	14	15	59	499	54.7	6.47
IPI00549189.4	Thimet oligopeptidase	126.14	42.82	2	27	27	56	689	78.8	6.05
IPI00967269.1	Uncharacterized protein	159.55	48.82	8	12	12	55	254	29.2	6.76
IPI00027745.4	Isoform Long of Beta-glucuronidase	142.77	27.50	9	16	16	53	651	74.7	7.02
IPI00218728.4	Isoform 1 of Platelet-activating factor acet	112.41	44.39	5	14	14	53	410	46.6	7.37
IPI00010346.1	Neurolysin, mitochondrial	120.37	43.47	6	30	30	51	704	80.6	6.64
IPI00013698.3	N-acylsphingosine amidohydrolase (Acid c	99.88	22.71	4	16	16	51	546	60.6	8.81
IPI00029997.1	6-phosphogluconolactonase	119.55	67.05	1	15	15	50	258	27.5	6.05
IPI00479018.3	Isoform 2 of Syntenin-1	147.01	47.81	7	8	8	49	297	32.3	7.53
IPI00293655.3	ATP-dependent RNA helicase DDX1	124.98	25.14	3	18	18	48	740	82.4	7.23
IPI00375426.8	Uncharacterized protein	105.47	38.08	3	9	9	47	323	36.2	8.10
IPI00472887.3	Isoform 2 of Cytoskeleton-associated prot	83.60	11.41	4	19	19	37	1972	218.4	8.06
IPI00026260.1	Isoform 1 of Nucleoside diphosphate kinas	78.60	59.21	9	4	8	37	152	17.3	8.41
IPI00465186.3	Isoform 2 of Receptor-type tyrosine-prote	72.79	14.54	17	17	17	35	1898	211.6	6.42
IPI00924935.1	cDNA FLJ57106, highly similar to Transfer	68.69	30.63	3	18	18	31	679	75.9	6.81
IPI01012178.1	Ceroid-lipofuscinosis neuronal protein 5	66.21	26.26	3	9	9	31	358	41.5	7.40
IPI00030320.4	Probable ATP-dependent RNA helicase DD	46.49	20.08	1	10	10	31	483	54.4	8.66
IPI00010949.3	Isoform 1 of Sialate O-acetylesterase	52.47	26.20	2	12	12	30	523	58.3	7.33
IPI00257882.7	Xaa-Pro dipeptidase	87.83	32.66	3	10	10	29	493	54.5	6.00
IPI00003815.3	Rho GDP-dissociation inhibitor 1	56.30	30.39	3	7	7	28	204	23.2	5.11
IPI00241860.4	Mitochondrial intermediate peptidase	48.84	27.63	1	17	17	27	713	80.6	7.05
IPI01010585.1	cDNA FLJ58994, highly similar to Collagen	60.31	17.57	5	6	6	26	404	43.6	6.89
IPI00301459.2	Group XV phospholipase A2	57.55	27.18	4	8	8	26	412	46.6	6.73
IPI00943894.1	glycogen phosphorylase, liver form isoforn	49.89	23.74	6	15	18	26	813	93.1	7.30
IPI00015856.6	aspartyl aminopeptidase	43.63	25.98	12	10	10	26	485	53.4	7.58
IPI00029605.1	N-acetylgalactosamine-6-sulfatase	68.60	32.57	2	14	14	24	522	58.0	6.74
IPI00465044.2	Protein RCC2	37.51	36.78	1	15	15	23	522	56.0	8.78
IPI00844513.1	Similar to Chain , Heat-Shock Cognate 70k	52.73	21.21	1	1	5	22	231	25.6	7.61
IPI00027438.2	Flotillin-1	51.00	37.70	19	12	12	22	427	47.3	7.49
IPI00013452.11	Bifunctional aminoacyl-tRNA synthetase	45.23	10.12	1	11	11	22	1512	170.5	7.33
IPI00746165.2	Isoform 1 of WD repeat-containing protein	41.75	39.77	7	14	14	22	606	66.2	6.65
IPI00220342.5	N(G),N(G)-dimethylarginine dimethylamin	67.87	48.42	4	10	10	21	285	31.1	5.81
IPI00646689.1	Thioredoxin domain-containing protein 17	39.11	54.47	1	5	5	21	123	13.9	5.52
IPI01014863.1	Acetyl-CoA acetyltransferase, cytosolic	53.88	23.17	2	7	7	20	397	41.3	6.92
IPI00305978.4	Aflatoxin B1 aldehyde reductase member	42.26	27.58	8	6	6	20	359	39.6	7.17
IPI00246975.8	Glutathione S-transferase Mu 3	30.23	32.89	2	5	6	20	225	26.5	5.54
